# Joint associations of parental personality traits and socio‐economic position with trajectories of offspring depression: Findings from up to 6925 families in a UK birth cohort

**DOI:** 10.1002/jcv2.12028

**Published:** 2021-08-22

**Authors:** Tim Cadman, Alex S. F. Kwong, Paul Moran, Heather O’Mahen, Iryna Culpin, Deborah A. Lawlor, Rebecca M. Pearson

**Affiliations:** ^1^ Integrative Epidemiology Unit (IEU) University of Bristol Bristol UK; ^2^ Population Health Science Bristol Medical School University of Bristol Bristol UK; ^3^ Division of Psychiatry University of Edinburgh Edinburgh UK; ^4^ Centre for Multilevel Modelling School of Education University of Bristol Bristol UK; ^5^ Population Health Science Centre for Academic Mental Health Bristol Medical School University of Bristol Bristol UK; ^6^ Bristol NIHR Biomedical Research Centre Bristol UK; ^7^ Department of Psychology College of Life and Environmental Sciences University of Exeter Exeter UK

**Keywords:** ALSPAC, intergenerational transmission, maternal mental health, offspring depression, personality

## Abstract

**Background:**

Parental personality may influence the course of offspring depression but epidemiological evidence for associations is lacking. It is also unknown whether associations between parental personality and offspring depression differ by socio‐economic position (SEP). Our aims were to describe the trajectories of depressive symptoms across adolescence of offspring of parents with and without maladaptive personality traits and to test for effect modification by SEP.

**Methods:**

A longitudinal study in the Avon Longitudinal Study of Parents and Children birth cohort (*n*s = 3054–7046). Exposures were binary measures of maladaptive maternal and paternal personality traits. The outcome was depressive symptoms measured over nine occasions (ages 11–24) using the short mood and feelings questionnaire (SMFQ; range: 0–26). Effect modifiers were parental education and self‐reported material hardship. Multilevel growth curve models were used to estimate trajectories.

**Results:**

offspring of mothers with high (vs. low) maladaptive traits showed higher levels of depressive symptoms at multiple ages of adolescence, the greatest of which was observed at age 22 (predicted SMFQ difference age 10 = 0.66, 95% confidence intervals [CIs]: 0.25 to 1.28; age 22 = 1.00, CI: 0.51 to 1.50). There was weaker and inconsistent evidence of an association between paternal maladaptive personality and offspring depressive symptoms (SMFQ difference age 10 = 0.21, CI: −0.58 to 0.99; age 22 = 0.02, CI: −0.94 to 0.90). Lower SEP was also associated with higher offspring depressive symptoms (SMFQ difference material hardship vs. no hardship age 10 = 0.79, 95% CI: 0.46 to 1.13; age 22 = 0.96, CI: 0.56 to 1.36). There was minimal statistical evidence for effect modification.

**Conclusions:**

The offspring of mothers with high levels of maladaptive personality traits show evidence of greater depressive symptoms throughout adolescence although the absolute increase in symptoms is small. Evidence for the associations with fathers' personality was weaker. Socio‐economic position and maladaptive personality traits appear to be independent risk factors for offspring depressive symptoms.

**Key points**

- Previous studies have shown that the offspring of mothers with maladaptive personality traits show elevated levels of depression measured at a single time point.
- We show how depressive symptoms develop throughout adolescence for offspring of parents with these traits.
- We find that from ages 15 to 20 symptoms increase more quickly in this group compared to offspring whose parents don't have these traits.
- We also show that socio‐economic position and parental personality are independent risk factors for offspring depression.
- From both a policy and clinical perspective, this study highlights the need for early interventions for offspring of parents with maladaptive personality traits which take account of the personality characteristics of the parents.

## INTRODUCTION

There is a large body of evidence showing that parental mental health problems are associated with an increase in the risk of a range of offspring mental health problems (Stein et al., 2014). The majority of the research has focused on outcomes associated with parental depression, especially in the perinatal period (Goodman et al., 2011). However, emerging evidence suggests that other parental traits such as maladaptive personality traits may also represent important risk factors for offspring mental health (Pearson et al., 2018).

Maladaptive personality traits include domains such as emotional dysregulation, suspicion, hostility and impulsivity (Pearson et al., 2018). While continuously distributed (Hopwood et al., 2018), at the extreme end these traits cluster together in the clinical syndromes described as borderline and antisocial personality disorders. These traits are moderately stable and inflexible self and behavioural characteristics and may affect offspring mental health development in the absence of other mental health problems (Eyden et al., 2016). Findings from the cross‐sectional studies of parents with borderline personality disorder (BPD) have reported the elevated levels of depressive symptoms in adolescent offspring (Abela et al., 2005; Barnow et al., 2006). Recently, in a well‐powered longitudinal study, we reported the existence of associations between maladaptive maternal personality traits and elevated levels of depression, anxiety and self‐harm in their offspring at age 18 (Pearson et al., 2018). We found a dose–response relationship, whereby the strongest associations with offspring mental health were where parents scored highly in multiple personality domains.

The mechanisms leading to elevated depressed symptoms in offspring of parents with maladaptive personality traits are likely to include both genetic and environmental factors. Heritability of BPD is estimated at 40% (Amad et al., 2014), and there is evidence that depression and maladaptive personality traits share common genetic correlates (Witt et al., 2017). In terms of environmental mechanisms, a key area of focus has been on parenting and the early attachment relationship. Parenting is a highly demanding and stressful task, requiring perspective taking and the regulation of one's own emotions in the service of the child's well‐being. Parents with maladaptive personality traits may become more quickly emotionally dysregulated and be more likely to respond with less optimal strategies (Eyden et al., 2016). Indeed, a number of cross‐sectional studies have reported parenting differences in this population, including higher levels of parental hostility (Herr et al., 2008), intrusiveness (Hobson et al., 2009), lower sensitivity (Crandell et al., 2003; Newman et al., 2007) and overprotectiveness among mothers with BPD (Elliot et al., 2014; Feldman et al., 1995). One longitudinal study also found that self‐report of overprotective and rejecting parenting styles partially mediated the relationship between mother and offspring BPD symptoms, although the size of the effect was modest (Reinelt et al., 2014).

### Limitations of previous research

Most studies examining the mental health of offspring of parents with high levels of these personality traits are cross‐sectional (Eyden et al., 2016), and the few longitudinal studies have only assessed offspring outcomes at only one time point (Pearson et al., 2018). However, it is important to understand how parental personality impacts the manifestation of depressed mood *over time*, to inform when and how children may be most at risk. For example, it is important to understand whether such traits influence the overall levels of depression at any time, or alternatively whether they are associated with steeper rises in depressed mood during sensitive periods in the life course, such as during adolescence (Crone & Dahl, 2012). Understanding the nature of this relationship could be important for developing critically timed interventions for individuals with parents with personality difficulties.

A further limitation is that few studies have explored whether contextual factors – such as socio‐economic position (SEP) – affect the relationship between parent and child mental health. A stated World Health Organisation objective is to better understand differential vulnerability to stressors for children from disadvantaged backgrounds (Blas et al., 2010). Understanding the relationship between parental personality and offspring depression in different social contexts could help to identify the most at‐risk groups (Mikkonen et al., 2016).

It is theoretically plausible that parental personality and offspring mental health could interact to affect child mental health. For example, parental traits such as impulsivity (e.g., financial impulsivity) could have a significantly more adverse impact on child mental health where the family has fewer financial and social resources. Previous research on the relationship between parental SEP and offspring depressive trajectories has consistently found that low SEP is associated with higher offspring depression (Costello et al., 2008; Ferro et al., 2015; Wickrama et al., 2009), with a recent children of twin study finding that this association is largely attributable to shared genetic risk. Two other studies reported an association in the same direction which did not reach the level of statistical significance (Ellis et al., 2017; Weeks et al., 2014), in the case of Ellis et al. very likely due to low power.

Evidence that SEP as an effect modifier of associations between parental mental health and offspring depression is mixed. For example, in our previous work, maternal education appeared to moderate the association between maternal postnatal depressive symptoms and offspring depression but not that of antenatal depression (Pearson et al., 2013). Similarly, a meta‐analysis, which included 65,619 mother–offspring pairs from 121 studies, reported that the relationship between maternal depression (at any point after birth) and offspring internalising in childhood was strongest in lower socio‐economic samples (Goodman et al., 2011). However, there was significant heterogeneity in effect sizes between studies in the high and low SEP groups, and it was not reported how consistently interaction effects were found within studies. Contrary to these findings, a large (*N* = 138,559 mother–offspring pairs) cohort study published since this meta‐analysis found maternal depression (experienced between child ages 9 and 14) and low SEP to be independent risk factors for later offspring depression (measured ages 15–20) with no statistical evidence of an interaction (Mikkonen et al., 2016). The lack of consistent evidence for SEP modifying the association of maternal depression with offspring depression may be due to different time periods when maternal depression is measured or because the small number of studies finding evidence of interaction is chance findings.

Finally, few studies have examined the association of paternal maladaptive personality traits with child outcomes. Paternal associations with offspring outcomes are important because (with the exception of sex‐linked effects) fathers and mothers contribute equally to genetic risk, yet may differ in caregiving and roles (McKinney & Renk, 2007). Evidence for the association of paternal perinatal depression with offspring depression is inconsistent. While some studies have observed positive associations of paternal depression with offspring mental health (Kvalevaag et al., 2013; Ramchandani et al., 2005), others found no evidence of an association or reported weaker associations in comparison with maternal depression (Pearson et al., 2013; Stein et al., 2014). We previously found a close to null association of maladaptive paternal personality with offspring anxiety, depression and self‐harm measured once when the offspring were 18 years old (Pearson et al., 2018); herein, we extend this research by exploring the associations of maternal and paternal maladaptive personality traits on offspring depression symptoms trajectories from childhood to early adulthood. The aims of this study were as follows:

1. To describe separately the trajectories of depressive symptoms of offspring of mothers and fathers with high versus low levels of maladaptive personality traits.
2. To describe the trajectories of depressive symptoms of offspring of mothers and fathers from low versus high SEP.
3. To test whether parental SEP is an effect modifier of the association between parental maladaptive personality traits and offspring depressive symptoms.

## METHOD

### Sample

The sample comprised participants from the Avon Longitudinal Study of Parents and Children (ALSPAC), an ongoing population‐based study. The study website contains details of all data available through a fully searchable data dictionary and variable search tool (http://www.bristol.ac.uk/alspac/researchers/our‐data/). Ethical approval for the study was obtained from the ALSPAC Ethics and Law Committee and the Local Research Ethics Committees (B3222). Informed consent for the use of data collected via questionnaires and clinics was obtained from participants following the recommendations of the ALSPAC Ethics and Law Committee at the time. Pregnant women resident in Avon, UK with expected dates of delivery from 1 April 1991 to 31 December 1992 were invited to take part in the study. The initial number of pregnancies enrolled is 14,541 (for these at least one questionnaire has been returned or a ‘Children in Focus’ clinic had been attended by 19 July 1999). Of these initial pregnancies, there were a total of 14,676 foetuses, resulting in 14,062 live births and 13,988 children who were alive at 1 year of age (for further details on the cohort profile, representativeness and phases of recruitment, see Boyd et al., 2013; Fraser et al., 2013; Northstone et al., 2019).

### Measures

#### Exposure: Parental maladaptive personality traits

Potentially maladaptive personality traits in mothers and fathers were assessed using the Karolinska Scales of Personality (KSP) inventory (Gustavsson, 1997) when their child was at a mean age of 9 years. The KSP is a self‐report questionnaire containing 135 questions measuring 15 personality traits relevant to psychological functioning and vulnerability to psychiatric conditions. We focused on five subscales identified a priori (Monotony Avoidance, Impulsivity, Verbal Anger, Suspicion and Detachment, number of items = 47) which are theoretically distinct from the neuroticism domain which links to depression. Rather, we focus on domains reflective of relational and affect dysregulation which are traits that differentiate personality disorders (which are of interest here) from depression and anxiety disorders. We derived a binary variable to indicate the presence or absence of maladaptive personality traits. Parents were categorised as having high maladaptive personality if they scored in the top quartile for at least three of the five selected scales. We took this approach because it is the combination of symptoms/traits that are important for personality disorder diagnoses and that are theorised to underlie disruption to relationships and parenting rather than any one trait alone. In addition, we have shown in our previous work that this binary indicator was a stronger predictor of later offspring mental health risk than continuous individual scores (Pearson et al., 2018).

#### Outcome: Depressive symptoms

Depressive symptoms were assessed using the short mood and feelings questionnaire (SMFQ) (Angold et al., 1995) on up to nine occasions between the ages of 11 and 24 years. The SMFQ is a 13‐item questionnaire that measures the occurrence of depressive symptoms over the preceding 2 weeks with higher scores indicating more severe depressive symptoms (range: 0–26). Questionnaires were completed by the offspring either via postal/Internet questionnaires or by computer at a clinic visit.

#### Potential effect modifiers: Socio‐economic position

Two indicators were chosen to capture different aspects of SEP: (i) education and (ii) material hardship.

Parental education is strongly related to future income and employment and also reflects non‐material family resources (e.g., knowledge) (Galobardes et al., 2006). Binary variables for maternal and paternal education were created from parents' report of their highest educational qualification at the time of the mothers' recruitment in early pregnancy. High education was defined as Advanced Level (A‐level) or comparable qualifications (A‐levels are exams taken in up to four subjects usually at age 18 years and required for university entry and some skilled jobs) or University degree. Low education was defined as no qualifications or qualifications below A‐level.

Material hardship was assessed within the first year of the child's life for both parents using the question: ‘How difficult at the moment do you find it to afford these items for the child: food, clothing, heating, rent, items for child’. Possible responses ranged from 0 (‘not difficult’) to 3 (‘very’), giving a total range of 0–15 across the five items. Binary variables were created for each parent created using a cut‐off of ≥5 corresponding to material hardship scores in the top 20% of the sample (Joinson et al., 2017). This aspect of SEP has previously been shown in ALSPAC to be associated with offspring depression (Joinson et al., 2017).

#### Confounders

Confounders were selected a priori on the basis that they were known to, or plausibly, influence parental maladaptive personality traits and offspring depression: parental age at child birth (years), maternal drinking in period of pregnancy (yes/no), parental depression (yes/no) during the postnatal period taken as the average score on the Edinburgh Postnatal Depression Scale measured at 2 and 8 months postpartum ≥13 as used in previous studies (Stein et al., 2014), smoking in pregnancy/at the time of partners pregnancy (yes/no) and self‐report of experiencing intimate partner violence (yes/no). We also adjusted for offspring sex as it is associated with depression and this adjustment may have improved the precision of our results.

## DATA ANALYSIS

### Trajectories of offspring depressive symptoms

#### Model specification

Trajectories of depressive symptoms were estimated using multilevel growth‐curve modelling with maximum likelihood (Bryk & Raudenbush, 1987; Steele, 2008). Briefly, multilevel growth‐curve models estimate population‐averaged trajectories that quantify how a trait changes over time. Descriptive statistics and previous research indicated that depressive symptoms followed a non‐linear pattern (Kwong et al., 2019), so we used a quartic polynomial model to account for this non‐linearity. The trajectory models were comprised of an intercept term and four age terms (age, age^2^, age^3^ and age^4^). All terms were allowed to freely vary (i.e., a random intercept and random slope model). Further information regarding model equations, diagnostic checks, along with tests examining the variation in the number of polynomials are reported in Tables S1–S3 and Figure S1.

#### Main effects of parental personality and SEP

To examine trajectories stratified by maternal and paternal maladaptive personality traits, we first created binary dummy variables to indicate the presence of maternal and paternal maladaptive personality traits and interacted these with all fixed effect terms (intercept and four age terms). Interpreting raw coefficients with multiple age terms can be complex; therefore, to interpret these differences, we compared predicted depressive symptoms scores for offspring exposed and unexposed to maladaptive parental personality traits at ages 10, 14, 18 and 22. Separate models were used to estimate the effect of maternal and paternal personality. To estimate the main effects of SEP, we used the same approach described here with binary variables indicating low versus high SEP. Full details are provided in Supporting Information S1.

### Effect modification of parental maladaptive personality traits association with offspring depression by SEP

Dummy variables for each level of the interaction between parental personality and each SEP indicator (exposed + high SEP, exposed + low SEP, not exposed + high SEP and not exposed + low SEP). These dummy variables were then interacted with the fixed effect terms as above. Effect modification was tested by comparing the difference in depressive symptoms for offspring exposed and unexposed to maladaptive parental personality within strata of high and low SEP at four key ages (10, 14, 18 and 22). Multilevel modelling was conducted using Stata 15 (StataCorp) using the user‐written runmlwin command (Leckie & Charlton, 2013), which calls the stand‐alone multilevel modelling package MLwiN v3.02 (www.cmm.bristol.ac.uk/MLwiN). All figures were plotted using the ggplot2 package in R version 3.51.

### Missing data

We included individuals with at least one measurement of depressive symptoms and complete data for maladaptive personality, which consisted of 7046 mother–child pairs and 3054 father–child pairs.

Available data for depressive symptoms decreased over time: 47% of the study sample had non‐missing data at age 11 which decreased to 25% by age 24 (Table S4). Missing depression data were handled using full information maximum likelihood estimation (FIML). Briefly, this assumes that the probability of an individual missing a measure of depressive symptoms does not depend on their underlying depressive symptoms score at that occasion, given their observed depressive symptoms trajectory at other occasions. Missing exposure data (i.e., parental personality, education and hardship) were handled using inverse probability weighting (IPW), which weights regression models for non‐response.

Tables S5 and S6 compare descriptive statistics for full ALSPAC sample and the maternal and paternal study samples. There is evidence that the study sample was of higher average SEP than the full ALSPAC sample; for example, the percentage of mothers with high education was 45% in the study sample versus 25% in the full ALSPAC sample.

## RESULTS

Table 1 shows sample characteristics of mothers and fathers with low and high levels of maladaptive personality traits. 15% of mothers and 11% of fathers were categorised as having high levels of maladaptive traits. Similar patterns were observed for both: Mothers and father with high trait levels were more likely to have lower education, experienced financial problems, smoked in/around the time of their partner's pregnancy and experienced intimate partner violence. They also both showed higher levels of depression in the postnatal period. We observed a low correlation between maladaptive maternal and paternal personality (*r* = 0.10).

**TABLE 1:** Sample characteristics of mothers and fathers

	Low maternal maladaptive traits		High maternal maladaptive traits		Odds ratio (95% CI)/mean difference
	Mean (SD)	*N* (%)	Mean (SD)	*N* (%)	
Mothers	*N* = 6002 (85%)		*N* = 1044 (15%)		
High education		2584 (0.46)		356 (0.37)	0.71 (0.62–0.82)
Material hardship		778 (0.14)		364 (0.07)	1.11 (1.09–1.13)
Age	29.35 (4.42)		28.37 (4.92)		−0.98
Drinking in pregnancy		3180 (0.56)		564 (0.59)	1.13 (0.98–1.30)
Smoking in pregnancy		874 (0.15)		279 (0.29)	2.26 (1.93–2.64)
Depression in postnatal period		391 (0.07)		175 (0.19)	3.04 (2.51–3.70)
Experienced partner violence		209 (0.04)		91 (0.10)	2.81 (2.18–3.64)
Fathers	*n* = 2726 (89%)		*n* = 328 (11%)		
High education		1560 (0.63)		139 (0.51)	0.60 (0.47–0.77)
Material hardship		391 (0.17)		165 (0.07)	1.15 (1.11–1.19)
Age	31.04 (6.63)		30.01 (7)		−1.03
Smoking in pregnancy		581 (0.25)		107 (0.41)	2.09 (1.61–2.72)
Depression in postnatal period		58 (0.02)		26 (0.1)	4.43 (2.74–7.18)
Experienced partner violence		70 (0.03)		22 (0.08)	3.04 (1.85–4.99)

*Note*: Descriptive data shown for participants with complete data for exposure (parental personality) and at least one depression outcome (SMFQ at one time point). Odds ratios represent probability of given characteristic for high versus low maladaptive personality group. Mean differences represent mean score for high maladaptive personality group minus mean score for low maladaptive personality group.

Abbreviation: SMFQ, short mood and feelings questionnaire.

### Aim 1: To describe the trajectories of depressive symptoms of offspring of mothers and fathers with high versus low levels of maladaptive personality traits

**FIGURE 1:**
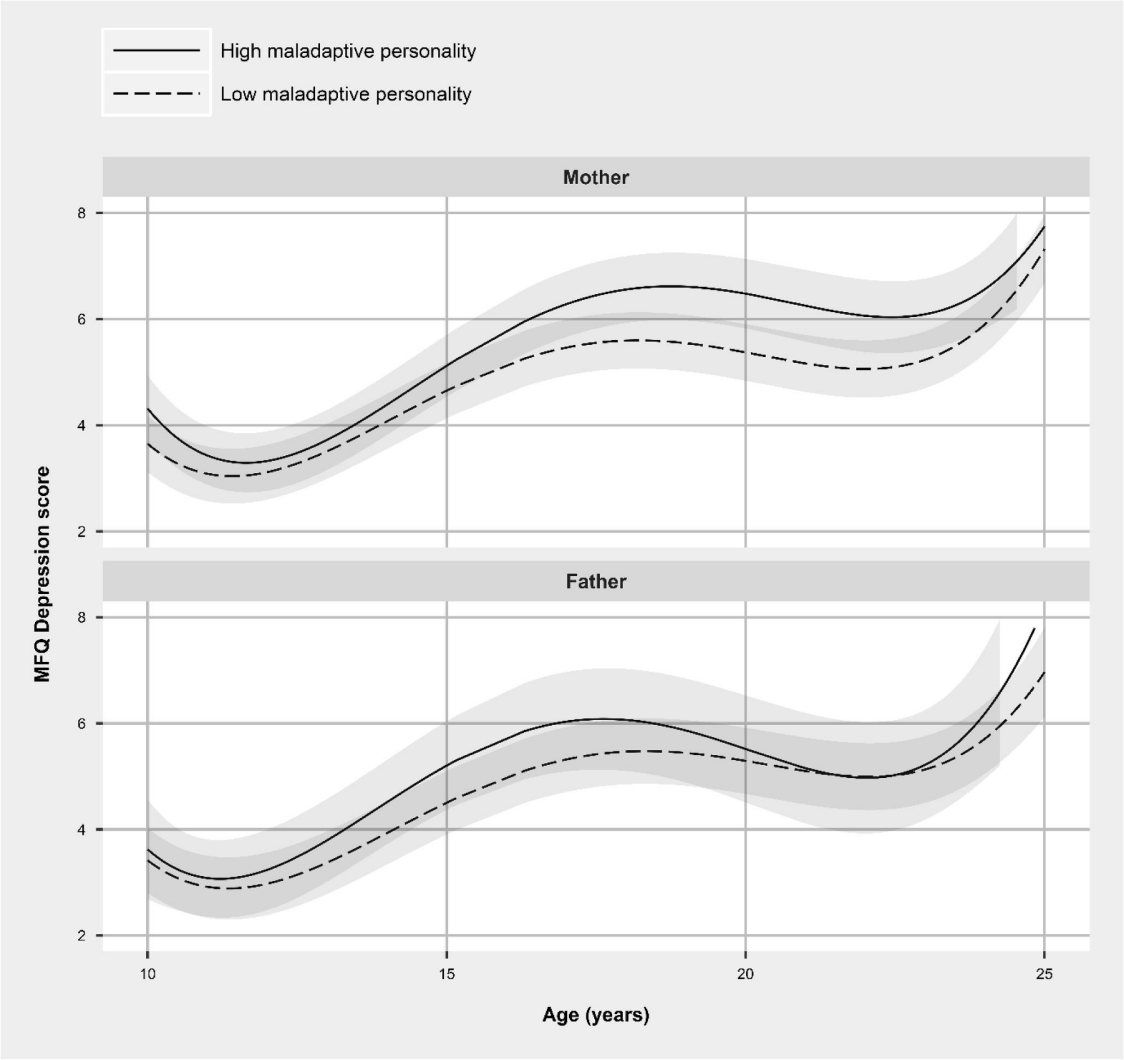
Trajectories of depressive symptoms of offspring by the presence or absence of high maladaptive personality traits in mothers (*N* = 6232) and fathers (*N* = 2098). Trajectories adjusted for following covariates: child sex, maternal age, drinking in pregnancy, smoking in pregnancy, postnatal depression score and physical partner violence. Of the *N* = 7046 mothers *n* = 1044 (14.8%) had high maladaptive personality traits, and of the *N* = 3054 fathers *n* = 328 (10.7%) had high maladaptive traits. Shaded areas represent 95% confidence intervals

Figure 1 shows the trajectories of depressive symptoms of offspring of mothers and fathers with and without high levels of maladaptive personality traits. Full model results are presented in Tables S7–S10. The shape of the trajectories of offspring exposed and unexposed to parental maladaptive personality traits and in relation to each parent was similar, with increase in depressive symptoms at age 17–18 years followed by a slower decline at age 22–23 years. At both the ends of the trajectories, there were short ‘upturns’ but these may be influenced by sparse data at these ages.

For mothers, there was evidence that the presence of maladaptive personality traits was associated with higher levels of offspring depressive symptoms across the trajectory from age 11 to 24 (predicted SMFQ difference in mean comparing those whose mothers had maladaptive personality traits to those who did not: age 10 = 0.66, 95% CI: 0.25 to 1.28; age 14 = 0.31, CI: 0.02 to 0.61; age 18 = 0.96, 95% CI: 0.56 to 1.37; age 22 = 1.00, CIs: 0.51 to 1.50).

Associations of paternal maladaptive personality traits with the trajectories of offspring depressive symptoms were weaker and close to null by age 22 (predicted difference age 10 = 0.21, 95% CI: −0.58 to 0.99; age 14 = 0.58, CI: −0.01 to 1.17; age 18 = 0.60, CI: −0.21 to 1.41; age 22 = 0.02, CI: −0.94 to 0.90); however, it should be noted that the paternal sample size is considerably smaller than that for maternal analyses and the confidence intervals for the two trajectories overlapped.

### Aim 2: To describe the trajectories of depressive symptoms of offspring of mothers and fathers from low versus high SEP

**FIGURE 2:**
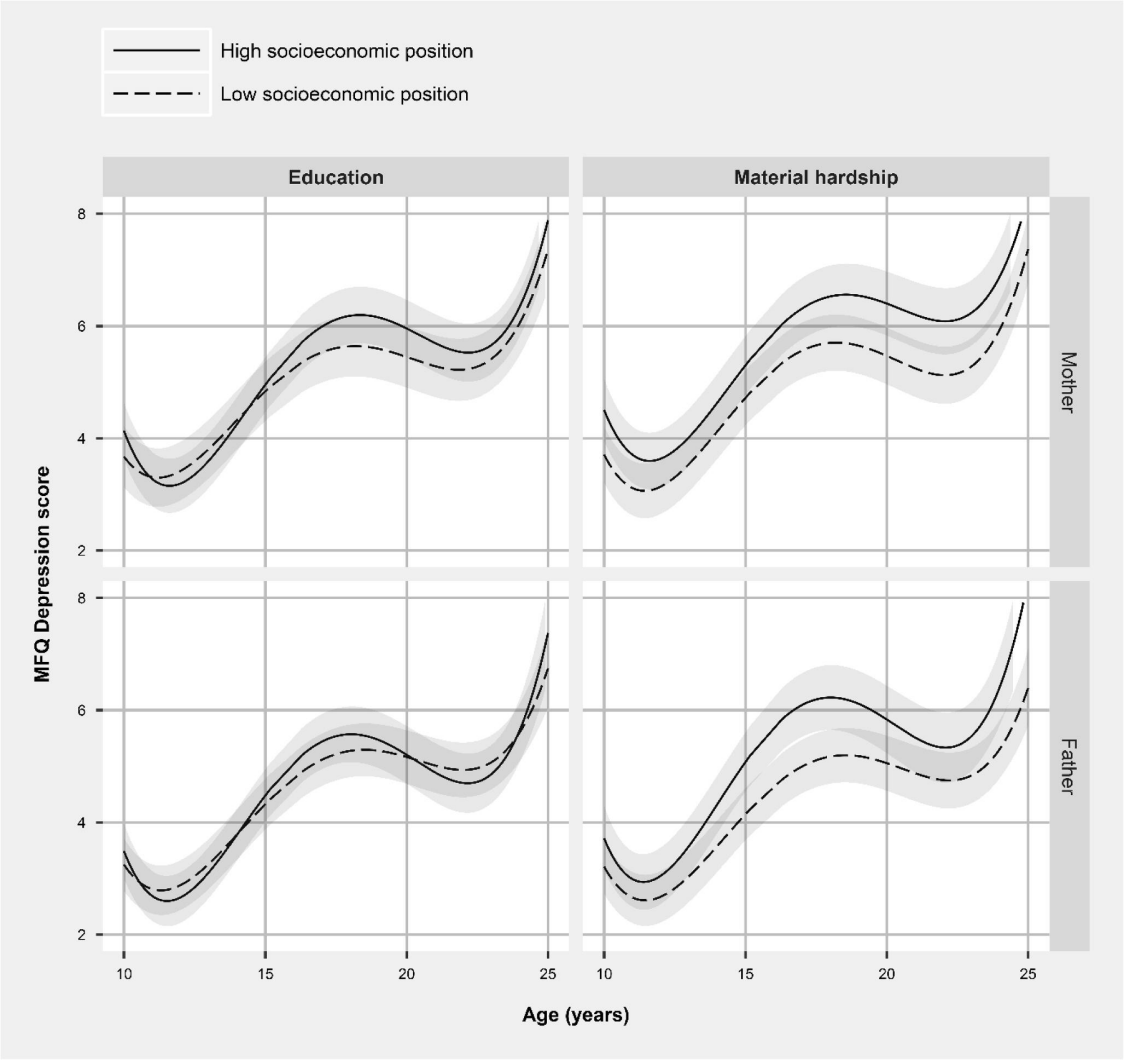
Trajectories of depressive symptoms stratified by parental socio‐economic position (*N*s = 3734–7577)

Figure 2 shows the offspring depressive trajectories stratified by the level of parental education and self‐reported material hardship. Results showed that the offspring of parents of lower SEP generally showed higher levels of depressive symptoms throughout adolescence. These associations were strongest for maternal and paternal self‐reported material hardship (predicted SMFQ difference maternal hardship vs. no hardship age 10 = 0.79, 95% CI: 0.46 to 1.13; age 14 = 0.51, CI: 0.28 to 0.75; age 18 = 0.83, CI: 0.50 to 1.15; age 22 = 0.96, CI: 0.56 to 1.36; fathers' age 10 = 0.79; CI: 0.46 to 1.13, age 14 = 0.51; CI: 0.28 to 0.75; age 18 = 0.83, CI: 0.50 to 1.15; age 22 = 0.96, CI: 0.56 to 1.36). A similar pattern was observed for parental education, although differences were smaller in magnitude (predicted SMFQ difference low versus. high education mothers age 10 = 0.46, 95% CI: 0.19 to 0.73; age 14 = −0.07, CI: −0.27 to 0.12; age 18 = 0.54, CI: 0.28 to 0.80; age 22 = 0.31, CI: 0.00 to 0.62; SMFQ difference by father education age 10 = 0.24, CI: −0.11 to 0.59; age 14 = −0.01, CI: −0.26 to 0.24; age 18 = 0.30, CI: −0.04 to 0.63; age 22 = −0.23, CI: −0.64 to 0.17). Full results are shown in Tables S11–S18.

### Aim 3: To examine whether associations of parental personality on offspring depression are moderated by SEP

**FIGURE 3:**
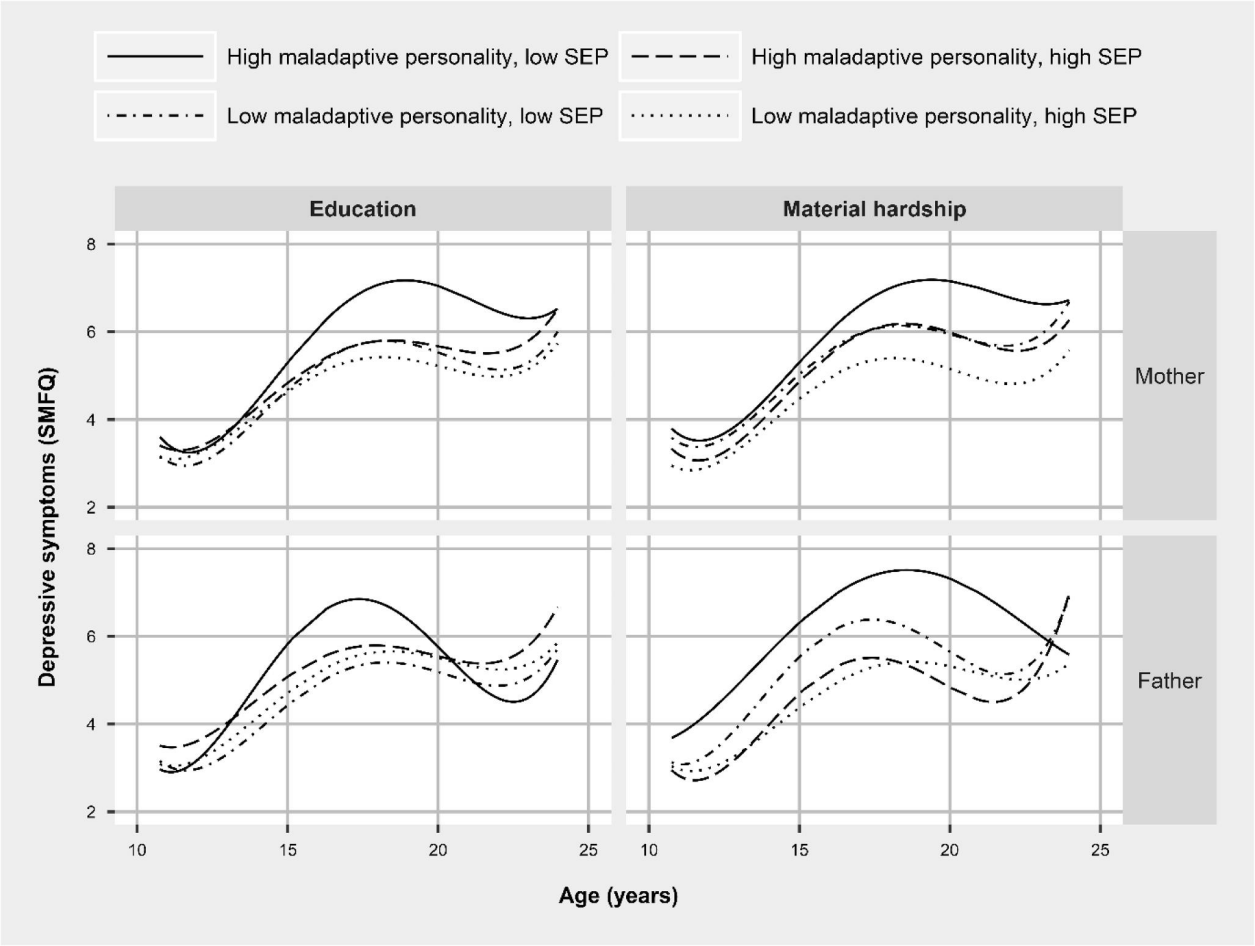
Trajectories of depressive symptoms stratified by parental personality and socio‐economic position (SEP). Trajectories adjusted for following covariates: child sex, maternal age, drinking in pregnancy, smoking in pregnancy, postnatal depression score and physical partner violence. Confidence intervals not shown due to graph scale. SMFQ, short mood and feelings questionnaire

To test whether the associations between maternal personality and offspring depressive symptoms were moderated by SEP, two separate analyses were completed for maternal education × personality in one model and material hardship × personality in a second model. The shape of the trajectories across strata was similar with increase in depressive symptoms for all groups at age 17–18 followed by a decline at age 22–23. For both indicators of SEP, there was evidence that at age 18 the highest levels of depressive symptoms were in the high maladaptive personality × low SEP groups; however, at ages 10 and 24, scores were very similar for all groups (Figure 3 and Tables S19–S22).

Effect modification was tested by comparing the predicted difference in depressive symptoms for offspring exposed and unexposed to maladaptive parental personality within strata of high and low SEP at four key ages (10, 14, 18 and 22). We found minimal statistical evidence for an interaction between SEP and either maternal or paternal personality, with only 2 of the 16 tests reaching the threshold of statistical significance (Table 2). For example, at age 10, the predicted difference in mean SMFQ comparing those exposed to maladaptive maternal personality to those who were not was 0.42 (−0.23 to 1.07) in those whose mothers had higher education and 0.67 (0.13 to 1.20) in those whose mothers had lower education, *p*‐interaction = .56.

**TABLE 2:** Difference in depressive symptoms comparing offspring exposed and unexposed to maladaptive parental adaptation within strata of high and low SEP

	Predicted difference in mean SMFQ (95% CI) high versus low maladaptive personality			Predicted difference in mean SMFQ (95% CI) high versus low maladaptive personality		
	High education	Low education	*p*‐Value	Low material hardship	High material hardship	*p*‐Value
Maternal						
Age 10	0.42 (−0.23 to 1.07)	0.67 (0.13 to 1.20)	.56	0.66 (0.16 to 1.16)	0.28 (−0.50 to 1.06)	.48
Age 14	0.16 (−0.30 to 0.61)	0.43 (0.04 to 0.81)	.38	0.26 (−0.10 to 0.61)	0.16 (−0.38 to 0.70)	.56
Age 18	0.37 (−0.26 to 1.00)	1.28 (0.75 to 1.82)	.03	0.76 (0.27 to 1.25)	0.86 (0.10 to 1.63)	.83
Age 24	0.55 (−0.19 to 1.29)	1.32 (0.66 to 1.99)	.13	0.76 (0.17 to 1.35)	1.10 (0.15 to 2.04)	.56
Paternal						
Age 10	0.42 (−0.64 to 1.48)	−0.24 (−1.52 to 1.05)	.44	0.29 (−0.70 to 1.27)	−0.05 (−1.46 to 1.37)	.95
Age 14	0.41 (−0.38 to 1.20)	1.07 (0.11 to 2.02)	.30	0.86 (−0.20 to 1.92)	0.70 (−0.58 to 1.99)	.28
Age 18	0.15 (−0.91 to 1.21)	1.38 (0.06 to 2.69)	.15	0.08 (−0.92 to 1.09)	1.15 (−0.35 to 2.64)	.25
Age 24	0.18 (−1.00 to 1.35)	−0.29 (−1.85 to 1.27)	.72	−0.44 (−1.54 to 0.66)	1.38 (−0.44 to 3.20)	.09

Abbreviations: CI, confidence interval; SEP, socio‐economic position; SMFQ, short mood and feelings questionnaire.

All findings were very similar when analyses were repeated using IPW (Tables S23–S27).

## DISCUSSION

Knowledge about the influence of parental personality on offspring mental health is limited. We found that the presence of maladaptive personality traits among mothers was associated with higher levels of offspring depressive symptoms across the trajectory from age 11 to 24. Yet, the shape of the trajectories of offspring exposed to maternal and paternal maladaptive personality traits was similar, with increase in depressive symptoms at age 17–18 years followed by a slower decline at age 22–23 years. The associations of paternal maladaptive personality traits with the trajectories of offspring depressive symptoms were weaker and close to null by age 22, although the paternal sample size was smaller than that for the maternal analyses.

We found that the highest levels of depressive symptoms were for offspring of mothers with both high levels of maladaptive traits and low SEP. However, there was no statistical evidence for an interaction suggesting that these risk factors are independent from one another and the greater risk observed in offspring exposed to both maladaptive personality and low education occurs due to the additive accumulation of two factors.

### Trajectories of depressive symptoms

The offspring of mothers with high levels of maladaptive personality traits had trajectories that were higher across adolescence and young adulthood, with the differences increasing from approximately age 15 and the greatest difference occurring around the age of 22. The magnitude of these effects was small, equating to a difference in SMFQ scores of approximately 1 point at age 22. This compliments and extends our previous findings that clinical levels of depression at 18 are more frequent among those exposed to maladaptive maternal personality traits (Pearson et al., 2018). It also suggests that adolescence is a period of high risk for this group of offspring, as symptoms do not necessarily decrease to population average levels.

In contrast, we found a smaller effect of father's personality on the level of offspring depressive symptoms. However, it is important to note that the sample size for paternal trajectory analysis was smaller and that for all estimates the confidence intervals for associations between maternal and paternal personality and offspring depression overlapped.

There is evidence that depression and maladaptive personality traits share common genetic correlates (Witt et al., 2017); therefore, part of the association between maternal maladaptive personality and offspring depression could be accounted for by shared genotype. It is also likely that some of the association between parental personality and offspring depression is mediated through environmental factors. This is supported by our findings of a weaker association of paternal personality with offspring depression, as if the effect was entirely genetic we would expect to see equivalent associations with maternal and paternal personality. Although not tested in this study, a plausible mediating factor for the association is through the parent–child relationship. This would be consistent with evidence of less sensitive parenting in mothers with higher levels of maladaptive personality traits (Eyden et al., 2016) and the prospective association between parenting style and offspring depression (Stein et al., 2014). Given the period of the study sample, it is likely that on average mothers would have had greater parenting responsibilities than fathers. This greater exposure could therefore explain the stronger associations observed between maternal (vs. paternal) maladaptive personality and offspring depression.

It was also possible that there was reverse or reciprocal causality, whereby traits or behaviours in the offspring elicit harsher behaviours in the parents. One mechanism could be via evocative gene–environment correlations: given that personality traits are partly heritable (Distel et al., 2011), offspring of parents with more difficult and maladaptive personality traits could be more likely to have such traits themselves. Having an impulsive and aggressive child, for example, may evoke more hostile parental responses which in turn might lead to unhappiness in the child.

If the finding of a stronger effect of maternal versus paternal personality on offspring depression is replicated, it could be explained by cultural differences in maternal and paternal parenting and/or quantity of time spent with mothers due to breastfeeding, higher frequency of non‐working or part‐time working mothers versus fathers in the United Kingdom at the time of the study. However, the notion of differential effects of mothers and father parenting on the child is increasingly questioned, given the limited empirical evidence that the parenting of mothers and fathers is qualitatively different (Cabrera et al., 2011; Fagan et al., 2014). Future studies should therefore attempt to replicate these findings of a weaker effect of paternal versus maternal personality on offspring depression and test whether any association between parental personality and offspring depression is mediated by parenting, relational factors or other indirect effects of traits such as impulsivity (e.g., a more chaotic home environment).

### Effect modification by SEP

We found consistent evidence that low parental SEP was associated with higher levels of offspring depression. These results fit with those from another large longitudinal study which found associations between maternal education and trajectories of offspring depression (Costello et al., 2008). While we also found that the highest levels of offspring depressive symptoms were in the high maladaptive traits, low SEP groups, there was minimal statistical evidence of effect modification. This is consistent with a recent large study which found independent associations between maternal depression and SEP on offspring depression but no evidence for effect modification (Mikkonen et al., 2016), but not with a previous ALSPAC study which did report an interaction between maternal postnatal depression and SEP on offspring depression (Pearson et al., 2013). These inconsistencies may be a result of the large sample required to detect interaction effects but also suggest that if there is an interaction effect it is likely a very small one (Kendler & Gardner, 2010).

The absence of effect modification is potentially an important clinical finding. It suggests that while both SEP and maladaptive parental personality are risk factors for offspring depression, the effect of parental personality traits such as high impulsivity or suspicion are the same across low and high SEP families. This finding was consistent using two separate indicators representing separate (though likely overlapping) aspects of SEP (Galobardes et al., 2006). This implies that there is unlikely to be a clinically unique group of ‘at‐risk’ parents with low SEP and high levels of maladaptive traits which require targeting. Instead, interventions are likely to be required on different levels, for example, policy and economic interventions to reduce inequality versus family and individual level interventions for parents with maladaptive personality traits.

### Summary and implications

This study has provided converging evidence that the offspring of mothers with high levels of maladaptive personality traits show a greater increase in depressive symptoms through adolescence, although the magnitude of the effect modest. A similar trend was observed for fathers but to a lesser degree, notwithstanding the considerably smaller paternal sample for the trajectory analysis. Thus, this describes two vulnerable groups: (i) mothers with high levels of maladaptive traits, many of whom have experienced interpersonal violence and mental health problems themselves (Moran et al., 2016), and (ii) their offspring who are at increased risk of depression throughout adolescence. If replicated these results may point to the potential need for early interventions for these families which take account of the interpersonal style and personality characteristics of the parents.

### Strengths and limitations

This was a large prospective cohort study with extensive repeated self‐report data on depressive symptoms. Extensive data on potential confounders were available and included in the analysis. However, it is still likely that residual confounding was present as traits such as personality and SEP indicators will be associated with a wide range of other individual and social factors. A key limitation of this study was the smaller sample size for the paternal trajectories compared to the maternal analysis, which limits the opportunity for comparison of associations between parents. A further limitation of the findings is the high attrition rate within ALSPAC, which is associated with sex and socio‐economic factors (Howe et al., 2013). However, our use of FIML and IPW (as shown in Tables S23–S27) would have partially mitigated this bias. While our use of polynomial models allowed the estimation of natural growth curves, interpretation of age coefficients was difficult and future studies modelling mental health trajectories could consider alternative approaches such as fractional polynomials or splines (Howe et al., 2016). In addition, notwithstanding the reasonable sample size, cell numbers for some of the interaction levels were still small and standard errors high. Using a binary variable to indicate personality traits may have reduced power and loses variation from the individual scores; however, this approach was chosen based on clinical and previous evidence that the accumulation of traits is most relevant to maladaptive personality. Finally, it should be acknowledged that differential reliability in the exposure across the moderator may have created artificial moderation.

### Future directions

These findings need to be replicated in an independent larger sample, to confirm whether there are differences in associations between mothers and fathers with maladaptive traits and offspring depressive symptoms. To understand the potential mechanisms of transmission, potential mediating pathways of the association should be tested (e.g., later parent–child relationships or parental depression), and potential genetic effects, including evocative, also explored.

## CONFLICT OF INTERESTS

The authors declared that there is no conflict of interest.

## AUTHOR CONTRIBUTIONS

Tim Cadman: Conceptualisation, Formal analysis, Project administration, Writing – original draft; Alex S. F. Kwong: Conceptualisation, Formal analysis, Methodology, Writing – review & editing; Paul Moran: Methodology, Writing – review & editing; Heather O'Mahen: Methodology, Writing – review & editing; Iryna Culpin: Methodology, Writing – review & editing; Deborah A. Lawlor: Methodology, Writing – review & editing; Rebecca M. Pearson: Conceptualisation, Data curation, Funding acquisition, Methodology, Project administration, Supervision, Writing – review & editing.

## ETHICAL APPROVAL

Ethical approval for the study was obtained from the Avon Longitudinal Study of Parents and Children Ethics and Law Committee and the Local Research Ethics Committees (B3222).

## Supporting information

Supporting Information S1

## Data Availability

The data that support the findings of this study are available from the Avon Longitudinal Study of Parents and Children. Restrictions apply to the availability of these data, which were used under license for this study. Data are available at http://www.bristol.ac.uk/alspac/researchers/access/ with the permission of Avon Longitudinal Study of Parents and Children.
