# Supplementary material for: Joint associations of parental personality traits and socio‐economic position with trajectories of offspring depression: Findings from up to 6925 families in a UK birth cohort
Source: JCPP Adv. 2021 Aug 22;1(3):e12028. doi: 10.1002/jcv2.12028 (PMC10242948; doi:10.1002/jcv2.12028)
Supplement: Supplementary file 1 — Supporting Information S1 [file JCV2-1-e12028-s001.docx]

**Supporting information**

**Electronic Methods**

Model fit for the trajectories were assessed using likelihood ratio tests and model fit statistics. We started by building a stepwise model which tested which model was preferred. The quadratic model was preferred to the linear model (*x*^2^ = 1383.27, p <.001), the cubic model was preferred to the quadratic model (*x*^2^ = 394.1, p <.001), and the quartic model preferred to the cubic model (*x*^2^ = 858.25, p <.001). Model fit statistics included: deviance, Akaike information criterion (AIC) and Bayesian information criterion (BIC), as recommended by (Singer & Willett, 2003). Briefly, lower deviance, AIC and BIC indicate better model fit. We examined model fit three times: first we examined model fit for just those with at least one assessment of the SMFQ (Table S1). Next, we examined model fit for individuals with at least one SMFQ assessment and data on maternal personality, maternal hardship and maternal education (Table S2). Finally, we examined model fit for individuals with at least one SMFQ assessment and data on paternal personality, paternal hardship and paternal education (Table S3). Both analyses indicted that a quartic polynomial model fitted the data the best. As a final check, we plotted all four models (linear, quadratic, cubic and quartic) over the descriptive data to visually compare the models. This once again indicated that that the quartic model was preferred (Figure S1).

| **Table S1. Comparisons between polynomial models with SMFQ data (n=9,399).** | | | |
| --- | --- | --- | --- |
| **Model** | **Deviance** | **AIC** | **BIC** |
| Linear Model | 254892.8 | 254904.8 | 254957 |
| Quadratic Polynomial | 252509.6 | 253529.6 | 253616.5 |
| Cubic Polynomial | 253115 | 253145.5 | 253275.8 |
| **Quartic Polynomial** | **252257.2** | **252299.2** | **252481.7** |

Model fit by the four polynomial models. SMFQ: short mood and feelings questionnaire; AIC: Akaike information criterion; BIC: Bayesian information criterion

| **Table S2. Comparisons between polynomial models with maternal personality, maternal hardship and maternal education data (n=6,428).** | | | |
| --- | --- | --- | --- |
| **Model** | **Deviance** | **AIC** | **BIC** |
| Linear Model | 193407.72 | 193419.7 | 193470.2 |
| Quadratic Polynomial | 192324.97 | 192345 | 192429.2 |
| Cubic Polynomial | 191997.62 | 192027.6 | 192153.9 |
| **Quartic Polynomial** | **191363.17** | **191405.2** | **191582** |

Model fit by the four polynomial models. SMFQ: short mood and feelings questionnaire; AIC: Akaike information criterion; BIC: Bayesian information criterion

| **Table S3. Comparisons between polynomial models with paternal personality, paternal hardship and paternal education data (n=2,445).** | | | |
| --- | --- | --- | --- |
| **Model** | **Deviance** | **AIC** | **BIC** |
| Linear Model | 82161.19 | 82173.2 | 82218.64 |
| Quadratic Polynomial | 81583.11 | 81603.11 | 81678.85 |
| Cubic Polynomial | 81402.64 | 81432.64 | 81546.25 |
| **Quartic Polynomial** | **81119.58** | **81161.58** | **81320.63** |

Model fit by the four polynomial models. SMFQ: short mood and feelings questionnaire; AIC: Akaike information criterion; BIC: Bayesian information criterion

**
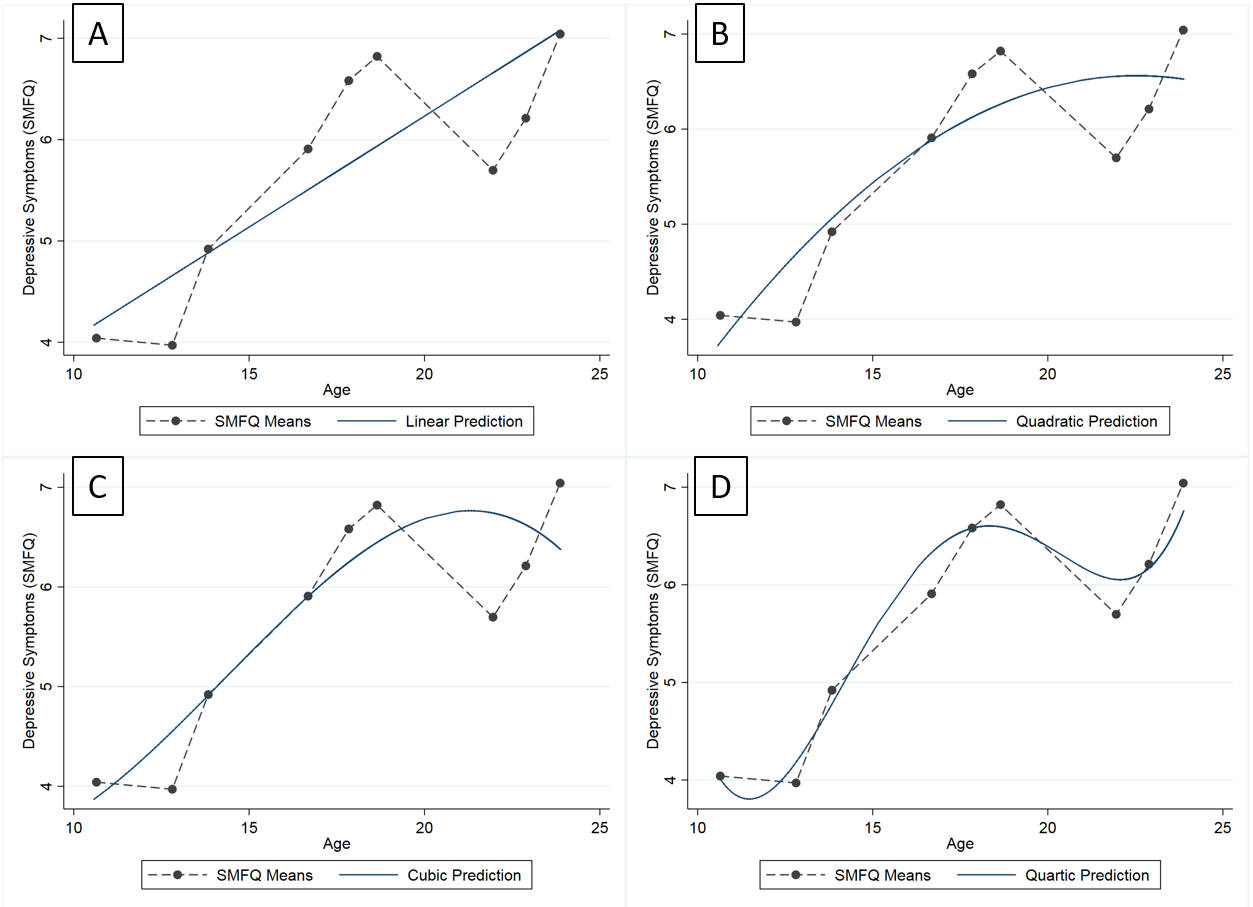
Figure S1. Comparisons between linear (A), quadratic (B), cubic (C) and quartic (D) models.**

**Model equations**

The simplest trajectory model consists of an intercept and slope. For this particular trajectory, the slope is comprised of 4 age terms: age, age^2^, age^3^ and age^4^. Thus, the trajectory in the simplest form can be denoted in the following equation:

$$y_{ij}=\beta_{0}+\beta_{1}t_{ij}+\beta_{2}t_{ij}^{2}+\beta_{3}t_{ij}^{3}+\beta_{4}t_{ij}^{4}+u_{0j}+u_{1j}t_{ij}+u_{2j}t_{ij}^{2}+u_{3j}t_{ij}^{3}+u_{4j}t_{ij}^{4}+e_{ij}$$

(Supplementary equation 1)

where $y_{ij}$ is the depressive symptom score and $t_{ij}$ is the age (centred around 18 years, to coincide with the end of adolescence to improve convergence) for individual $j$ at occasion $i$. $t_{ij}^{2}$, $t_{ij}^{3}$, and $t_{ij}^{4}$, represent the quadratic, cubic and quartic age terms respectively and $u_{0j}$, $u_{1j}$, $u_{2j}$, $u_{3j}$ and $u_{4j}$ are the random intercept, linear, quadratic, cubic and quartic effects, respectively, and $e_{ij}$ is the occasion-specific residual. The model is therefore set up to predict an average population trajectory from the fixed effects ($\beta_{0}$ - $\beta_{4})$, whilst allowing everyone to have their own trajectory that deviates from the population-averaged intercept and age functions ($u_{0j}$ - $u_{4j}$).

The random effects are assumed multivariate normal distributed with zero mean vector and constant covariance matrix:

$$\left( \begin{aligned} u_{0j} \\ u_{1j} \\ u_{2j} \\ u_{3j} \\ u_{4j} \end{aligned} \right)\sim N\left\{ \left( \begin{aligned} 0 \\ 0 \\ 0 \\ 0 \\ 0 \end{aligned} \right), \left( \begin{aligned} \sigma_{u0}^{2} \\ \sigma_{u01} \\ \sigma_{u02} \\ \sigma_{u03} \\ \sigma_{u04} \end{aligned}\begin{aligned} \\ \sigma_{u1}^{2} \\ \sigma_{u12} \\ \sigma_{u13} \\ \sigma_{u14} \end{aligned}\begin{aligned} \\ \\ \sigma_{u2}^{2} \\ \sigma_{u23} \\ \sigma_{u24} \end{aligned}\begin{aligned} \\ \\ \\ \sigma_{u3}^{2} \\ \sigma_{u34} \end{aligned}\begin{aligned} \\ \\ \\ \\ \sigma_{u4}^{2} \end{aligned} \right) \right\}$$

(Supplementary equation 2)

The elements of the covariance matrix summarise the degree to which individual-specific trajectories vary around the population-averaged trajectories. The residuals are assumed normally distributed with zero mean:

$$e_{ij}\sim N(0, \sigma_{e}^{2})$$

(Supplementary equation 3)

**Predicted Depressive Symptom Trajectories**

The predicted depressive symptom score for individual $j$ at occasion $i$ is given by:

$$\hat{y}_{ij}=\hat{\beta}_{0}+\hat{\beta}_{1}t_{ij}+\hat{\beta}_{2}t_{ij}^{2}+\hat{\beta}_{3}t_{ij}^{3}+\hat{\beta}_{4}t_{ij}^{4}+\hat{u}_{0j}+\hat{u}_{1j}t_{ij}+\hat{u}_{2j}t_{ij}^{2}+\hat{u}_{3j}t_{ij}^{3}+\hat{u}_{4j}t_{ij}^{4}+e_{ij}$$

(Supplementary equation 4)

The average predicted depressive symptom score at a given age $t_{ij}$ can be obtained by setting all random effects (e.g., $u_{0j}$) to 0.

**Association Between Parental Personality and Depressive Symptom Trajectories**

We ran sperate models to examine the association between maternal and paternal personality and trajectories of depressive symptoms. We used $x_{1j}$ to determine the presence of parental personality for both parents. When $x_{1j}$ is set to 1, the model produces a main effect of personality on the trajectories. When $x_{1j}$ is set to 0, the parameters containing $x_{1j}$ are not included. The depressive symptom score for individual $j$ at occasion $i$ is therefore given by:

$$y_{ij}=\beta_{0}+\beta_{1}t_{ij}+\beta_{2}t_{ij}^{2}+\beta_{3}t_{ij}^{3}+\beta_{4}t_{ij}^{4}+\beta_{5}x_{1j}+\beta_{6}x_{1j}t_{ij}+\beta_{7}x_{1j}t_{ij}^{2}+\beta_{8}x_{1j}t_{ij}^{3} +\beta_{9}x_{1j}t_{ij}^{4}$$

$$+u_{0j}+u_{1j}t_{ij}+u_{2j}t_{ij}^{2}+u_{3j}t_{ij}^{3} +u_{4j}t_{ij}^{4}$$

(Supplementary equation 5)

The average predicted depressive symptom score for those individuals with and without parental personality at a given age $t_{ij}$ can be obtained by setting all random effects to 0 and by setting $x_{1j}$ to 0 (no parental personality) or 1 (yes parental personality) accordingly.

**Association Between Parental Personality, SEP and Depressive Symptom Trajectories**

We ran sperate models to examine the association between the parental personality X varying SEP variables and trajectories of depressive symptoms. However, each model had the same equation and different SEP variables can substituted into the equation used here (e.g., just replace early financial problems with maternal education). First, we created 4 dummy variables to examine the association between the interaction of parental personality and the various indicators of SEP: with low personality X high SEP (0/0) as the baseline or reference trajectory, high personality X high SEP (1/0), high personality X low SEP (1/1) and low personality X low SEP (0/1). To derive the 3-way interaction between parental personality X SEP X trajectories of depressive symptoms, those dummy variables were then interacted with the four age terms (SEP indicator X maternal personality X intercept age, age^2^, age^3^ and age^4^). Each level of the interaction therefore contained 5 terms: the intercept and four age terms, and resulted in 4 individual trajectories, a low personality and high SEP trajectory, a high personality and high SEP trajectory, a high personality and low SEP trajectory, and a low personality and low SEP trajectory.

The model can be written in several ways. Firstly, we used $x_{j}$ to determine the presence of parental personality X SEP, so when $x_{0j}$ is set to 0, the model is indicative of the baseline trajectory (i.e., low personality X high SEP). When $x_{2j}$ is set to 1, the model is reflective of the high personality X high SEP group. When $x_{3j}$ is set to 2, the model is reflective of the high personality X low SEP group and when $x_{4j}$ is set to 3, the model is reflective of the low personality X low SEP group.

The depressive symptom score for this 3-way interaction for individual $j$ at occasion $i$ is therefore given by:

$$y_{ij}=\beta_{0}x_{0j}+\beta_{1}x_{0j}t_{ij}+\beta_{2}x_{0j}t_{ij}^{2}+\beta_{3}x_{0j}t_{ij}^{3}+\beta_{4}x_{0j}t_{ij}^{4}$$

$$+ \beta_{5}x_{1j}+\beta_{6}x_{1j}t_{ij}+\beta_{7}x_{1j}t_{ij}^{2}+\beta_{8}x_{1j}t_{ij}^{3} +\beta_{9}x_{1j}t_{ij}^{4}$$

$$+ \beta_{10}x_{2j}+ \beta_{11}x_{2j}t_{ij}+\beta_{12}x_{2j}t_{ij}^{2}+\beta_{13}x_{2j}t_{ij}^{3} +\beta_{14}x_{2j}t_{ij}^{4}$$

$$+ \beta_{15}x_{3j}+ \beta_{16}x_{3j}t_{ij}+\beta_{17}x_{3j}t_{ij}^{2}+\beta_{18}x_{3j}t_{ij}^{3} +\beta_{19}x_{3j}t_{ij}^{4}$$

$$+u_{0j}+u_{1j}t_{ij}+u_{2j}t_{ij}^{2}+u_{3j}t_{ij}^{3} +u_{4j}t_{ij}^{4}$$

(Supplementary equation 6)

The average predicted depressive symptom score for those individuals with or without parental personality X SEP at a given age $t_{ij}$ can be obtained by setting all random effects to 0 and by setting $x_{j}$ to 0 (low personality X high SEP), 1 (high personality X high SEP), 2 (high personality X low SEP) or 3 (personality X low SEP).

An alternative model parameterisation for the predicted population-average for each trajectory can be written as this:

Low pers X high SEP: $E\left( \hat{y}_{ij} | t_{ij}, x_{0j}=1, x_{1j}=0, x_{2j}=0, x_{3j}=0 \right)= \beta_{0}+\beta_{1}t_{ij}+\beta_{2}t_{ij}^{2}+\beta_{3}t_{ij}^{3}+\beta_{4}t_{ij}^{4}$
Low pers X high SEP: $E\left( \hat{y}_{ij} | t_{ij}, x_{0j}=0, x_{1j}=1, x_{2j}=0, x_{3j}=0 \right)= {(\beta}_{0}+\beta_{5}) +{(\beta}_{1}+ \beta_{6})t_{ij}+{(\beta}_{2}+ \beta_{7})t_{ij}^{2}+{(\beta}_{3}+ \beta_{8})t_{ij}^{3}+{(\beta}_{4}+ \beta_{9})t_{ij}^{4}$
High pers X low SEP: $E\left( \hat{y}_{ij} | t_{ij}, x_{0j}=0, x_{1j}=0, x_{2j}=1, x_{3j}=0 \right)= {(\beta}_{0}+\beta_{10}) +{(\beta}_{1}+ \beta_{11})t_{ij}+{(\beta}_{2}+ \beta_{12})t_{ij}^{2}+{(\beta}_{3}+ \beta_{13})t_{ij}^{3}+{(\beta}_{4}+ \beta_{14})t_{ij}^{4}$Low pers X low SEP: $E\left( \hat{y}_{ij} | t_{ij}, x_{0j}=0, x_{1j}=0, x_{2j}=0, x_{3j}=1 \right)= {(\beta}_{0}+\beta_{15}) +{(\beta}_{1}+ \beta_{16})t_{ij}+{(\beta}_{2}+ \beta_{17})t_{ij}^{2}+{(\beta}_{3}+ \beta_{18})t_{ij}^{3}+{(\beta}_{4}+ \beta_{19})t_{ij}^{4}$

**Supplementary results**

*Table S4: Available data on SMFQ depressive symptoms at each time point*

| **Measurement occasion** | **Age**  Mean (SD) | **SMFQ** **score**  Mean (SD) | **Available** **data**  N (%) | **Missing data**  N (%) |
| --- | --- | --- | --- | --- |
| 1 | 10.65 (0.26) | 4.04 (3.51) | 7364 (47.07) | 8281 (52.93) |
| 2 | 12.81 (0.23) | 3.97 (3.86) | 6716 (42.93) | 8929 (57.07) |
| 3 | 13.84 (0.21) | 4.92 (4.49) | 6019 (38.47) | 9626 (61.53) |
| 4 | 16.68 (0.24) | 5.91 (5.64) | 4997 (31.94) | 10648 (68.06) |
| 5 | 17.84 (0.4) | 6.58 (5.25) | 4497 (28.74) | 11148 (71.26) |
| 6 | 18.65 (0.49) | 6.82 (5.93) | 3335 (21.32) | 12310 (78.68) |
| 7 | 21.95 (0.52) | 5.70 (5.58) | 3305 (21.12) | 12340 (78.88) |
| 8 | 22.89 (0.52) | 6.21 (5.54) | 3856 (24.65) | 11789 (75.35) |
| 9 | 23.87 (0.52) | 7.04 (6.07) | 3887 (24.84) | 11758 (75.16) |

*Table S5: Comparison between maternal study sample and full ALSPAC sample*

|  | Full ALSPAC sample (N = 15, 645) | Study sample (N = 7,046) | Test of difference |
| --- | --- | --- | --- |
| High education (N, %) | 1470 (25.0) | 2940 (44.5) | <0.001 |
| Material hardship (N, %) | 1781 (31.2) | 1382 (21.4) | <0.001 |
| Age (mean, SD) | 26.88 (5.11) | 29.21 (4.51) | <0.001 |
| Drinking in pregnancy (N, %) | 3449 (52.6) | 3744 (56.4) | <0.001 |
| Smoking in pregnancy (N, %) | 2212 (33.0) | 1153 (17.3) | <0.001 |
| Experienced partner violence (N, %) | 321 (6.6) | 300 (4.6) | <0.001 |
| Depression in post-natal period (N, %) | 625 (12.0) | 566 (8.7) | <0.001 |
| Depressive symptoms T1 (mean, SD) | 4.30 (3.81) | 3.98 (3.44) | 0.003 |
| Depressive symptoms T2 (mean, SD) | 4.24 (4.16) | 3.91 (3.79) | 0.007 |
| Depressive symptoms T3 (mean, SD) | 4.94 (4.67) | 4.92 (4.45) | 0.863 |
| Depressive symptoms T4 (mean, SD) | 6.57 (6.16) | 5.79 (5.53) | 0.001 |
| Depressive symptoms T5 (mean, SD) | 6.87 (5.32) | 6.52 (5.23) | 0.076 |
| Depressive symptoms T6 (mean, SD) | 7.29 (6.20) | 6.74 (5.88) | 0.059 |
| Depressive symptoms T7 (mean, SD) | 6.42 (5.88) | 5.56 (5.51) | 0.001 |
| Depressive symptoms T8 (mean, SD) | 6.46 (5.81) | 6.16 (5.48) | 0.197 |
| Depressive symptoms T9 (mean, SD) | 7.71 (6.40) | 6.88 (5.97) | 0.001 |

*Table S6: Comparison between paternal study sample and full ALSPAC sample*

|  | Full ALSPAC sample (N = 15, 645) | Study sample (N = 7,046) | Test of difference |
| --- | --- | --- | --- |
| High education (N, %) | 0.45 (0.50) | 0.62 (0.49) | <0.001 |
| Material hardship (N, %) | 1378 (30.3) | 575 (22.2) | <0.001 |
| Age (mean, SD) | 30.17 (6.91) | 30.95 (6.67) | <0.001 |
| Smoking in pregnancy (N, %) | 2889 (41.9) | 688 (26.2) | <0.001 |
| Experienced partner violence (N, %) | 232 (4.1) | 92 (3.4) | 0.187 |
| Depression in post-natal period (N, %) | 214 (3.8) | 84 (3.2) | 0.174 |
| Depressive symptoms T1 (mean, SD) | 4.17 (3.67) | 3.81 (3.21) | <0.001 |
| Depressive symptoms T2 (mean, SD) | 4.07 (3.95) | 3.81 (3.70) | 0.009 |
| Depressive symptoms T3 (mean, SD) | 4.93 (4.49) | 4.91 (4.49) | 0.835 |
| Depressive symptoms T4 (mean, SD) | 6.10 (5.78) | 5.65 (5.43) | 0.005 |
| Depressive symptoms T5 (mean, SD) | 6.80 (5.36) | 6.26 (5.07) | 0.001 |
| Depressive symptoms T6 (mean, SD) | 7.07 (6.01) | 6.51 (5.80) | 0.007 |
| Depressive symptoms T7 (mean, SD) | 5.87 (5.80) | 5.48 (5.27) | 0.046 |
| Depressive symptoms T8 (mean, SD) | 6.25 (5.65) | 6.16 (5.38) | 0.62 |
| Depressive symptoms T9 (mean, SD) | 7.33 (6.26) | 6.60 (5.72) | <0.001 |

| *Table S7: Main Effects of Maternal Personality on SMFQ Trajectories* | | | | | | | | | | |
| --- | --- | --- | --- | --- | --- | --- | --- | --- | --- | --- |
|  | Unadjusted Model (n=7046) | | | | | Adjusted Model (n=6232) | | | | |
| Parameter | Estimate | Low 95% CIs | High 95% CIs | Std. Error | *p*-value | Estimate | Low 95% CIs | High 95% CIs | Std. Error | *p*-value |
| *β*0 - No Maternal Personality Intercept (ref) | 6.258 | 6.116 | 6.400 | 0.07 | <.001 | 5.583 | 5.055 | 6.112 | 0.27 | <.001 |
| *β*1 - No Maternal PersonalityxAge *(*ref*)* | 0.084 | 0.047 | 0.120 | 0.02 | <.001 | 0.071 | 0.033 | 0.110 | 0.02 | <.001 |
| *β*2 - No Maternal PersonalityxAge^2 (ref) | -0.094 | -0.104 | -0.085 | 0.005 | <.001 | -0.094 | -0.104 | -0.084 | 0.01 | <.001 |
| *β*3 - No Maternal PersonalityAge^3 (ref) | 0.004 | 0.003 | 0.005 | 0.001 | <.001 | 0.004 | 0.003 | 0.005 | 0.001 | <.001 |
| *β*4 - No Maternal PersonalityxAge^4 (ref) | 0.002 | 0.001 | 0.002 | 0.0001 | <.001 | 0.002 | 0.001 | 0.002 | 0.0001 | <.001 |
| *β*5 - Maternal Personality Intercept | 1.395 | 1.016 | 1.773 | 0.19 | <.001 | 0.939 | 0.536 | 1.343 | 0.21 | <.001 |
| *β*6 - Maternal PersonalityxAge | 0.101 | 0.002 | 0.199 | 0.05 | 0.046 | 0.133 | 0.026 | 0.240 | 0.05 | 0.015 |
| *β*7 - Maternal PersonalityxAge^2 | -0.033 | -0.059 | -0.007 | 0.01 | 0.012 | -0.021 | -0.049 | 0.007 | 0.01 | 0.138 |
| *β*8 - Maternal PersonalityxAge^3 | -0.001 | -0.004 | 0.001 | 0.001 | 0.335 | -0.003 | -0.006 | 0.001 | 0.002 | 0.103 |
| *β*9 - Maternal PersonalityxAge^4 | 0.0005 | -0.0001 | 0.001 | 0.0003 | 0.092 | 0.0002 | -0.0004 | 0.001 | 0.0003 | 0.462 |
| Deviance | 208010.07 | | | | | 186238.98 | | | | |
| *Adjusted for child sex, maternal age at birth, drinking in pregnancy, smoking in pregnancy, postnatal depression and physical abuse | | | | | | | |  |  |  |
| *Ref is the reference category which should be added to each category | | | |  |  |  |  |  |  |  |
| *Intercepts are at age 18 to coincide with the end of adolescence | |  |  |  |  |  |  |  |  |  |

| *Table S7A: Random effects of Maternal Personality on SMFQ Trajectories* | | | | | | | | |
| --- | --- | --- | --- | --- | --- | --- | --- | --- |
|  | Unadjusted Model | | | | Adjusted Model | | | |
| Parameter | Estimate | Std. Error | Low 95% CIs | High 95% CIs | Estimate | Std. Error | Low 95% CIs | High 95% CIs |
| var(Intercept) | 18.24201 | 0.488454 | 17.28466 | 19.19936 | 17.18713 | 0.493122 | 16.22063 | 18.15363 |
| cov(Intercept, Age) | 0.456653 | 0.086598 | 0.2869245 | 0.6263812 | 0.491146 | 0.089063 | 0.3165854 | 0.6657066 |
| var(Age) | 0.378913 | 0.028894 | 0.3222824 | 0.4355436 | 0.373128 | 0.030299 | 0.3137432 | 0.4325131 |
| cov(Intercept, Age^2) | -0.48984 | 0.026697 | -0.5421603 | -0.437512 | -0.4616 | 0.027221 | -0.5149489 | -0.4082454 |
| cov(Age, Age^2) | 0.022692 | 0.005615 | 0.0116866 | 0.0336979 | 0.019952 | 0.00583 | 0.0085244 | 0.0313791 |
| var(Age^2) | 0.027827 | 0.002198 | 0.023518 | 0.0321357 | 0.025851 | 0.002268 | 0.0214055 | 0.0302966 |
| cov(Intercept, Age^3) | 0.007716 | 0.002512 | 0.002793 | 0.0126392 | 0.005762 | 0.002599 | 0.0006683 | 0.0108552 |
| cov(Age, Age^3) | -0.00668 | 0.000777 | -0.0082079 | -0.0051613 | -0.00659 | 0.00082 | -0.0082009 | -0.0049861 |
| cov(Age^2, Age^3) | -0.00074 | 0.000168 | -0.0010742 | -0.0004143 | -0.00066 | 0.000176 | -0.0010022 | -0.0003117 |
| var(Age^3) | 0.000171 | 2.25E-05 | 0.000127 | 0.000215 | 0.00017 | 2.39E-05 | 0.000123 | 0.0002165 |
| cov(Intercept, Age^4) | 0.006346 | 0.000515 | 0.0053359 | 0.007355 | 0.005875 | 0.000527 | 0.0048419 | 0.006909 |
| cov(Age, Age^4) | -0.0006 | 0.000118 | -0.0008307 | -0.000368 | -0.00055 | 0.000123 | -0.0007966 | -0.000313 |
| cov(Age^2, Age^4) | -0.00038 | 4.33E-05 | -0.0004677 | -0.0002979 | -0.00035 | 4.49E-05 | -0.0004341 | -0.0002581 |
| cov(Age^3, Age^4) | 1.97E-05 | 3.53E-06 | 0.0000127 | 0.0000266 | 1.82E-05 | 3.72E-06 | 0.0000109 | 0.0000255 |
| var(Age^4) | 5.59E-06 | 9.04E-07 | 3.81E-06 | 7.36E-06 | 4.92E-06 | 9.43E-07 | 3.07E-06 | 6.76E-06 |

*Table S8: Maternal personality comparisons at Various Ages of Each Trajectory*

|  | Age 10 | Age 14 | Age 18 | Age 22 |
| --- | --- | --- | --- | --- |
| Predicted low maladaptive maternal personality score | 3.65 (3.12, 4.18) | 4.07 (3.55, 4.59) | 5.59 (5.06, 6.12) | 5.06 (4.52, 5.60) |
| Predicted high maladaptive maternal personality score | 4.31 (3.68, 4.94) | 4.38 (3.81, 4.98) | 6.56 (5.93, 7.19) | 6.06 (5.38, 6.74) |
| Difference, *P*-Value | 0.66 (0.25, 1.08), =.002 | 0.31 (0.01, 0.61), =.035 | 0.96 (0.56, 1.37), <.001 | 1.00 (0.51, 1.50), <.001 |
| SMFQ % Difference | 2.54 | 1.19 | 3.69 | 3.85 |

*Adjusted for child sex, maternal age at birth, drinking in pregnancy, smoking in pregnancy, postnatal depression and intimate partner violence

Values in parentheses represent 95% confidence intervals

| *Table S9: Main Effects of Paternal Personality on SMFQ Trajectories* | | | | | | | | | | |
| --- | --- | --- | --- | --- | --- | --- | --- | --- | --- | --- |
|  | Unadjusted Model (n=3054) | | | | | Adjusted Model (n=2098) | | | | |
| Parameter | Estimate | Low 95% CIs | High 95% CIs | Std. Error | *p*-value | Estimate | Low 95% CIs | High 95% CIs | Std. Error | *p*-value |
| *β*0 - No Paternal Personality Intercept (ref) | 6.196 | 5.999 | 6.394 | 0.10 | <.001 | 5.452 | 4.837 | 6.067 | 0.31 | <.001 |
| *β*1 - No Paternal PersonalityxAge *(*ref*)* | 0.101 | 0.051 | 0.150 | 0.03 | <.001 | 0.091 | 0.033 | 0.148 | 0.03 | 0.002 |
| *β*2 - No Paternal PersonalityxAge^2 (ref) | -0.087 | -0.101 | -0.074 | 0.01 | <.001 | -0.090 | -0.105 | -0.075 | 0.01 | <.001 |
| *β*3 - No Paternal PersonalityxAge^3 (ref) | 0.004 | 0.002 | 0.005 | 0.001 | <.001 | 0.004 | 0.002 | 0.005 | 0.001 | <.001 |
| *β*4 - No Paternal PersonalityxAge^4 (ref) | 0.002 | 0.001 | 0.002 | 0.0001 | <.001 | 0.002 | 0.001 | 0.002 | 0.0002 | <.001 |
| *β*5 - Paternal Personality Intercept | 0.590 | -0.046 | 1.225 | 0.32 | 0.069 | 0.625 | -0.182 | 1.431 | 0.41 | 0.129 |
| *β*6 - Paternal PersonalityxAge | -0.113 | -0.272 | 0.046 | 0.08 | 0.163 | -0.138 | -0.336 | 0.060 | 0.10 | 0.173 |
| *β*7 - Paternal PersonalityxAge^2 | -0.024 | -0.066 | 0.019 | 0.02 | 0.271 | -0.033 | -0.085 | 0.019 | 0.03 | 0.214 |
| *β*8 - Paternal PersonalityxAge^3 | 0.003 | -0.002 | 0.007 | 0.002 | 0.249 | 0.004 | -0.002 | 0.010 | 0.003 | 0.165 |
| *β*9 - Paternal PersonalityxAge^4 | 0.0004 | -0.0005 | 0.001 | 0.0004 | 0.34 | 0.001 | -0.0004 | 0.002 | 0.001 | 0.223 |
| Deviance | 98907.63 | | | | | 69046.23 | | | | |
| *Adjusted for child sex, paternal age at birth, drinking in pregnancy, smoking in pregnancy, postnatal depression and physical abuse | | | | | | | |  |  |  |
| *Ref is the reference category which should be added to each category | | | |  |  |  |  |  |  |  |
| *Intercepts are at age 18 to coincide with the end of adolescence | |  |  |  |  |  |  |  |  |  |

| *Table S9A: Random effects of Paternal Personality on SMFQ Trajectories* | | | | | | | | |
| --- | --- | --- | --- | --- | --- | --- | --- | --- |
|  | Unadjusted Model | | | | Adjusted Model | | | |
| Parameter | Estimate | Std. Error | Low 95% CIs | High 95% CIs | Estimate | Std. Error | Low 95% CIs | High 95% CIs |
| var(Intercept) | 17.72973 | 0.675538 | 16.4057 | 19.05376 | 17.11157 | 0.774345 | 15.59389 | 18.62926 |
| cov(Intercept, Age) | 0.419252 | 0.117651 | 0.1886596 | 0.6498438 | 0.550644 | 0.135144 | 0.2857669 | 0.81552 |
| var(Age) | 0.398347 | 0.039266 | 0.3213884 | 0.4753064 | 0.385382 | 0.045015 | 0.2971544 | 0.4736094 |
| cov(Intercept, Age^2) | -0.4862 | 0.036186 | -0.557122 | -0.4152767 | -0.4553 | 0.04064 | -0.5349553 | -0.37565 |
| cov(Age, Age^2) | 0.013771 | 0.007457 | -0.0008444 | 0.0283867 | 0.008168 | 0.008351 | -0.0082 | 0.0245349 |
| var(Age^2) | 0.026464 | 0.002901 | 0.020779 | 0.0321498 | 0.022358 | 0.003199 | 0.0160883 | 0.0286268 |
| cov(Intercept, Age^3) | 0.008676 | 0.003402 | 0.0020078 | 0.0153444 | 0.003955 | 0.003976 | -0.0038383 | 0.0117473 |
| cov(Age, Age^3) | -0.0072 | 0.001053 | -0.0092675 | -0.0051383 | -0.00735 | 0.001232 | -0.0097603 | -0.0049326 |
| cov(Age^2, Age^3) | -0.00059 | 0.000223 | -0.0010251 | -0.0001509 | -0.00039 | 0.000255 | -0.0008905 | 0.0001091 |
| var(Age^3) | 0.000187 | 3.05E-05 | 0.0001275 | 0.0002468 | 0.000204 | 3.64E-05 | 0.0001331 | 0.0002757 |
| cov(Intercept, Age^4) | 0.006494 | 0.000695 | 0.0051314 | 0.0078564 | 0.005665 | 0.000781 | 0.0041353 | 0.0071948 |
| cov(Age, Age^4) | -0.00043 | 0.000157 | -0.0007322 | -0.0001183 | -0.00037 | 0.000177 | -0.0007196 | -0.0000263 |
| cov(Age^2, Age^4) | -0.00037 | 5.71E-05 | -0.0004772 | -0.0002534 | -0.00027 | 6.29E-05 | -0.0003973 | -0.0001506 |
| cov(Age^3, Age^4) | 0.000017 | 4.69E-06 | 7.82E-06 | 0.0000262 | 1.54E-05 | 5.42E-06 | 4.73E-06 | 2.60E-05 |
| var(Age^4) | 5.30E-06 | 1.19E-06 | 2.97E-06 | 7.64E-06 | 3.55E-06 | 1.32E-06 | 9.59E-07 | 6.14E-06 |

*Table S10: Paternal personality comparisons at Various Ages of Each Trajectory*

|  | Age 10 | Age 14 | Age 18 | Age 22 |
| --- | --- | --- | --- | --- |
| Predicted low maladaptive paternal personality score | 3.42 (2.80, 4.03) | 3.93 (3.34, 4.52) | 5.47 (4.85, 6.08) | 4.99 (0.32) |
| Predicted high maladaptive paternal personality score | 3.63 (2.68, 4.57) | 4.51 (3.72, 5.31) | 6.06 (5.10, 7.03) | 4.97 (0.53) |
| Difference, *P*-Value | 0.21 (-0.58, 0.99) =.603 | 0.58 (-0.01, 1.17), =.053 | 0.60 (-0.21, 1.41), =.149 | 0.02 (0.47), =.967 |
| SMFQ % Difference | 0.81 | 2.23 | 2.30 | 0.08 |

*Adjusted for child sex, maternal age at birth, drinking in pregnancy, smoking in pregnancy, postnatal depression and intimate partner violence. Values in parentheses represent 95% confidence intervals

| *Table S11: Main Effects of Maternal Education at Birth on SMFQ Trajectories* | | | | | | | | | | |
| --- | --- | --- | --- | --- | --- | --- | --- | --- | --- | --- |
|  | Unadjusted Model (n=8449) | | | | | Adjusted Model (n=7577) | | | | |
| Parameter | Estimate | Low 95% CIs | High 95% CIs | Std. Error | *p*-value | Estimate | Low 95% CIs | High 95% CIs | Std. Error | *p*-value |
| *β*0 - Low Maternal Education Intercept (ref) | 6.867 | 6.698 | 7.036 | 0.09 | <.001 | 6.165 | 5.664 | 6.667 | 0.26 | <.001 |
| *β*1 - Low Maternal EducationxAge *(ref)* | 0.134 | 0.089 | 0.180 | 0.02 | <.001 | 0.121 | 0.074 | 0.168 | 0.02 | <.001 |
| *β*2 - Low Maternal EducationxAge^2 (ref) | -0.118 | -0.130 | -0.107 | 0.01 | <.001 | -0.116 | -0.129 | -0.104 | 0.01 | <.001 |
| *β*3 - Low Maternal EducationxAge^3 (ref) | 0.003 | 0.002 | 0.005 | 0.001 | <.001 | 0.004 | 0.002 | 0.005 | 0.001 | <.001 |
| *β*4 - Low Maternal EducationxAge^4 (ref) | 0.002 | 0.002 | 0.002 | 0.0001 | <.001 | 0.002 | 0.002 | 0.002 | 0.0001 | <.001 |
| *β*5 - Maternal Education Intercept | -0.678 | -0.928 | -0.428 | 0.13 | <.001 | -0.533 | -0.791 | -0.274 | 0.13 | <.001 |
| *β*6 - Maternal EducationxAge | -0.070 | -0.135 | -0.005 | 0.03 | 0.035 | -0.065 | -0.132 | 0.002 | 0.03 | 0.059 |
| *β*7 - Maternal EducationxAge^2 | 0.038 | 0.021 | 0.055 | 0.01 | <.001 | 0.036 | 0.019 | 0.054 | 0.01 | <.001 |
| *β*8 - Maternal EducationxAge^3 | 0.001 | -0.001 | 0.003 | 0.001 | 0.405 | 0.001 | -0.001 | 0.003 | 0.001 | 0.489 |
| *β*9 - Maternal EducationxAge^4 | -0.001 | -0.001 | -0.0003 | 0.0002 | 0.001 | -0.001 | -0.001 | -0.0003 | 0.0002 | 0.001 |
| Deviance | 232296.84 | | | | | 212791.96 | | | | |
| *Adjusted for child sex, maternal age at birth, drinking in pregnancy, smoking in pregnancy, postnatal depression and physical abuse | | | | | | | |  |  |  |
| *Ref is the reference category which should be added to each category  *Intercepts are at age 18 to coincide with the end of adolescence | | | |  |  |  |  |  |  |  |

| *Table S11A: Random effects of Maternal Education at Birth on SMFQ Trajectories* | | | | | | | | |
| --- | --- | --- | --- | --- | --- | --- | --- | --- |
|  | Unadjusted Model | | | | Adjusted Model | | | |
| Parameter | Estimate | Std. Error | Low 95% CIs | High 95% CIs | Estimate | Std. Error | Low 95% CIs | High 95% CIs |
| var(Intercept) | 18.84722 | 0.472973 | 17.92021 | 19.77423 | 17.43176 | 0.465836 | 16.51873 | 18.34478 |
| cov(Intercept, Age) | 0.430847 | 0.084917 | 0.2644133 | 0.5972813 | 0.433642 | 0.085066 | 0.2669152 | 0.6003682 |
| var(Age) | 0.401776 | 0.028622 | 0.3456786 | 0.4578737 | 0.395454 | 0.02929 | 0.3380468 | 0.4528614 |
| cov(Intercept, Age^2) | -0.50102 | 0.026005 | -0.5519843 | -0.4500481 | -0.46516 | 0.025936 | -0.5159928 | -0.4143253 |
| cov(Age, Age^2) | 0.025644 | 0.005532 | 0.014802 | 0.0364867 | 0.023482 | 0.00562 | 0.0124661 | 0.0344969 |
| var(Age^2) | 0.028098 | 0.002154 | 0.0238767 | 0.0323189 | 0.026417 | 0.002181 | 0.0221424 | 0.0306905 |
| cov(Intercept, Age^3) | 0.009446 | 0.002476 | 0.0045944 | 0.0142983 | 0.007633 | 0.002476 | 0.0027799 | 0.0124865 |
| cov(Age, Age^3) | -0.00733 | 0.000774 | -0.0088485 | -0.0058142 | -0.00721 | 0.000792 | -0.0087579 | -0.005654 |
| cov(Age^2, Age^3) | -0.00086 | 0.000167 | -0.0011834 | -0.0005289 | -0.00078 | 0.00017 | -0.0011073 | -0.000443 |
| var(Age^3) | 0.000193 | 2.25E-05 | 0.000149 | 0.000237 | 0.000187 | 0.000023 | 0.0001422 | 0.0002322 |
| cov(Intercept, Age^4) | 0.006631 | 0.000506 | 0.00564 | 0.0076225 | 0.006025 | 0.000504 | 0.0050378 | 0.0070129 |
| cov(Age, Age^4) | -0.00066 | 0.000117 | -0.000892 | -0.0004338 | -0.00062 | 1.19E-04 | -0.0008544 | -0.0003884 |
| cov(Age^2, Age^4) | -0.00039 | 4.27E-05 | -4.76E-04 | -0.0003084 | -3.61E-04 | 4.32E-05 | -4.46E-04 | -2.76E-04 |
| cov(Age^3, Age^4) | 2.22E-05 | 3.52E-06 | 1.53E-05 | 2.91E-05 | 2.05E-05 | 3.58E-06 | 1.34E-05 | 2.75E-05 |
| var(Age^4) | 5.84E-06 | 8.96E-07 | 4.09E-06 | 7.60E-06 | 5.26E-06 | 9.08E-07 | 3.48E-06 | 7.04E-06 |

*Table S12: Maternal education comparisons at various ages of each trajectory*

|  | Age 10 | Age 14 | Age 18 | Age 22 |
| --- | --- | --- | --- | --- |
| Predicted low maternal education score | 4.14 (3.63, 4.64) | 4.25 (3.77, 4.74) | 6.18 (5.68, 6.69) | 5.53 (5.02, 6.05) |
| Predicted high maternal education score | 3.67 (3.13, 4.22) | 4.32 (3.80, 4.85) | 5.64 (5.10, 6.18) | 5.22 (4.67, 5.78) |
| Difference, *P*-Value | 0.46 (0.19, 0.73), =.001 | -0.07 (-0.27, 0.12), =.046 | 0.54 (0.28, 0.80), <.001 | 0.31 (0, 0.62), .051 |
| SMFQ % Difference | 1.77 | 0.30 | 2.08 | 1.19 |

*Adjusted for child sex, maternal age at birth, drinking in pregnancy, smoking in pregnancy, postnatal depression and intimate partner violence

Values in parentheses represent 95% confidence intervals

| *Table S13: Main Effects of Paternal Education at Birth* | | | | | | | | | | |
| --- | --- | --- | --- | --- | --- | --- | --- | --- | --- | --- |
|  | Unadjusted Model (n=7626) | | | | | Adjusted Model (n=4159) | | | | |
| Parameter | Estimate | Low 95% CIs | High 95% CIs | Std. Error | *p*-value | Estimate | Low 95% CIs | High 95% CIs | Std. Error | *p*-value |
| *β*0 - Low Paternal Education Intercept (ref) | 6.702 | 6.508 | 6.896 | 0.10 | <.001 | 5.565 | 5.069 | 6.060 | 0.25 | <.001 |
| *β*1 - Low Paternal EducationxAge *(*ref*)* | 0.084 | 0.032 | 0.137 | 0.03 | 0.002 | 0.054 | -0.018 | 0.125 | 0.04 | 0.14 |
| *β*2 - Low Paternal EducationxAge^2 (ref) | -0.119 | -0.133 | -0.105 | 0.01 | <.001 | -0.121 | -0.140 | -0.103 | 0.01 | <.001 |
| *β*3 - Low Paternal EducationxAge^3 (ref) | 0.005 | 0.003 | 0.006 | 0.001 | <.001 | 0.005 | 0.003 | 0.007 | 0.001 | <.001 |
| *β*4 - Low Paternal EducationxAge^4 (ref) | 0.002 | 0.002 | 0.002 | 0.0001 | <.001 | 0.002 | 0.002 | 0.003 | 0.0002 | <.001 |
| *β*5 - Paternal Education Intercept | -0.404 | -0.663 | -0.146 | 0.13 | 0.002 | -0.307 | -0.644 | 0.030 | 0.17 | 0.074 |
| *β*6 - Paternal EducationxAge on SMFQ Trajectories | 0.015 | -0.053 | 0.083 | 0.03 | 0.671 | 0.054 | -0.036 | 0.143 | 0.05 | 0.238 |
| *β*7 - Paternal EducationxAge^2 | 0.034 | 0.016 | 0.052 | 0.01 | <.001 | 0.039 | 0.016 | 0.062 | 0.01 | 0.001 |
| *β*8 - Paternal EducationxAge^3 | -0.001 | -0.003 | 0.001 | 0.001 | 0.36 | -0.002 | -0.005 | 0.001 | 0.001 | 0.149 |
| *β*9 - Paternal EducationxAge^4 | -0.001 | -0.001 | -0.0002 | 0.0002 | 0.001 | -0.001 | -0.001 | -0.0003 | 0.0002 | 0.002 |
| Deviance | 213336.52 | | | | | 123171.59 | | | | |
| *Adjusted for child sex, paternal age at birth, drinking in pregnancy, smoking in pregnancy, postnatal depression and physical abuse | | | | | | | |  |  |  |
| *Ref is the reference category which should be added to each category  *Intercepts are at age 18 to coincide with the end of adolescence | | | |  |  |  |  |  |  |  |

| *Table S13A: Random effects of Paternal Education at Birth on SMFQ Trajectories* | | | | | | | | |
| --- | --- | --- | --- | --- | --- | --- | --- | --- |
|  | Unadjusted Model | | | | Adjusted Model | | | |
| Parameter | Estimate | Std. Error | Low 95% CIs | High 95% CIs | Estimate | Std. Error | Low 95% CIs | High 95% CIs |
| var(Intercept) | 18.1886 | 0.477488 | 17.25274 | 19.12446 | 16.67664 | 0.576017 | 15.54767 | 17.80562 |
| cov(Intercept, Age) | 0.377364 | 0.086164 | 0.2084867 | 0.5462419 | 0.4174 | 0.105192 | 0.2112263 | 0.6235728 |
| var(Age) | 0.409238 | 0.029345 | 0.3517224 | 0.4667529 | 0.423037 | 0.036581 | 0.3513408 | 0.4947338 |
| cov(Intercept, Age^2) | -0.48882 | 0.026371 | -0.5405101 | -0.4371388 | -0.45301 | 0.031802 | -0.5153443 | -0.3906832 |
| cov(Age, Age^2) | 0.027441 | 0.005667 | 0.0163336 | 0.0385491 | 0.02394 | 0.006917 | 0.0103837 | 0.0374971 |
| var(Age^2) | 0.028154 | 0.002195 | 0.023851 | 0.0324569 | 0.025683 | 0.002643 | 0.0205024 | 0.0308641 |
| cov(Intercept, Age^3) | 0.010125 | 0.002519 | 0.005188 | 0.0150624 | 0.007632 | 0.003078 | 0.0015991 | 0.013664 |
| cov(Age, Age^3) | -0.00776 | 0.000797 | -0.0093237 | -0.0061992 | -0.00837 | 0.000998 | -0.0103261 | -0.0064136 |
| cov(Age^2, Age^3) | -0.00091 | 0.000172 | -0.0012469 | -0.0005747 | -0.00084 | 0.00021 | -0.0012493 | -0.0004262 |
| var(Age^3) | 0.000208 | 2.32E-05 | 0.0001623 | 0.0002533 | 0.000226 | 2.92E-05 | 0.0001685 | 0.0002829 |
| cov(Intercept, Age^4) | 0.00649 | 0.000514 | 0.0054835 | 0.0074973 | 0.005897 | 0.000619 | 0.0046835 | 0.0071103 |
| cov(Age, Age^4) | -0.00073 | 0.00012 | -0.0009666 | -0.0004946 | -0.0007 | 1.48E-04 | -0.0009921 | -0.0004122 |
| cov(Age^2, Age^4) | -0.0004 | 4.37E-05 | -4.88E-04 | -0.0003165 | -3.61E-04 | 5.27E-05 | -4.65E-04 | -2.58E-04 |
| cov(Age^3, Age^4) | 2.42E-05 | 3.64E-06 | 1.71E-05 | 3.14E-05 | 2.39E-05 | 4.49E-06 | 1.51E-05 | 3.27E-05 |
| var(Age^4) | 6.25E-06 | 9.20E-07 | 4.44E-06 | 8.05E-06 | 5.61E-06 | 1.11E-06 | 3.43E-06 | 7.80E-06 |

*Table S14: Paternal education comparisons at various ages of each trajectory*

|  | Age 10 | Age 14 | Age 18 | Age 22 |
| --- | --- | --- | --- | --- |
| Predicted low paternal education score | 3.49 (3.0, 3.99) | 3.78 (3.32, 4.24) | 5.57 (5.07, 6.07) | 4.70 (4.17, 5.23) |
| Predicted high paternal education score | 3.25 (2.78, 3.72) | 3.78 (3.34, 4.23) | 5.27 (4.81, 5.74) | 4.94 (4.45, 5.42) |
| Difference, *P*-Value | 0.24 (-0.11, 0.59), =.173 | -0.01 (-0.26, 0.24), =.966 | 0.30 (-0.04, 0.63), =.087 | -0.23 (-0.64, 0.17), =.260 |
| SMFQ % Difference | 0.92 | 0.04 | 1.15 | 0.88 |

*Adjusted for child sex, maternal age at birth, drinking in pregnancy, smoking in pregnancy, postnatal depression and intimate partner violence

Values in parentheses represent 95% confidence intervals

| *Table S15: Main Effects of Maternal Hardship (MHA) on SMFQ Trajectories* | | | | | | | | | | |
| --- | --- | --- | --- | --- | --- | --- | --- | --- | --- | --- |
|  | Unadjusted Model (n=8230) | | | | | Adjusted Model (n=7412) | | | | |
| Parameter | Estimate | Low 95% CIs | High 95% CIs | Std. Error | *p*-value | Estimate | Low 95% CIs | High 95% CIs | Std. Error | *p*-value |
| *β*0 - No MHA Intercept (ref) | 6.345 | 6.203 | 6.486 | 0.072 | <.001 | 5.690 | 5.191 | 6.190 | 0.255 | <.001 |
| *β*1 - No MHAxAge (ref) | 0.086 | 0.050 | 0.123 | 0.019 | <.001 | 0.075 | 0.038 | 0.113 | 0.019 | <.001 |
| *β*2 - No MHAxAge^2 (ref) | -0.100 | -0.110 | -0.091 | 0.005 | <.001 | -0.098 | -0.108 | -0.088 | 0.005 | <.001 |
| *β*3 - No MHAxAge^3 (ref) | 0.004 | 0.003 | 0.005 | 0.001 | <.001 | 0.004 | 0.003 | 0.005 | 0.001 | <.001 |
| *β*4 - No MHAxAge^4 (ref) | 0.002 | 0.002 | 0.002 | 0.0001 | <.001 | 0.002 | 0.002 | 0.002 | 0.0001 | <.001 |
| *β*5 - MHA Intercept | 0.987 | 0.677 | 1.297 | 0.158 | <.001 | 0.813 | 0.489 | 1.138 | 0.166 | <.001 |
| *β*6 - MHAxAge | 0.069 | -0.014 | 0.153 | 0.042 | 0.103 | 0.074 | -0.013 | 0.162 | 0.045 | 0.097 |
| *β*7 - MHAxAge^2 | -0.008 | -0.029 | 0.014 | 0.011 | 0.488 | -0.007 | -0.030 | 0.016 | 0.012 | 0.536 |
| *β*8 - MHAxAge^3 | -0.001 | -0.003 | 0.001 | 0.001 | 0.408 | -0.001 | -0.004 | 0.001 | 0.001 | 0.419 |
| *β*9 - MHAxAge^4 | 0.0001 | -0.0003 | 0.001 | 0.0002 | 0.548 | 0.0001 | -0.0003 | 0.001 | 0.0002 | 0.582 |
| Deviance | 226369 | | | | | 208337 | | | | |
| *Adjusted for child sex, maternal age at birth, drinking in pregnancy, smoking in pregnancy, postnatal depression and physical abuse | | | | | | | | |  |  |
| *Ref is the reference category which should be added to each category  *Intercepts are at age 18 to coincide with the end of adolescence | | | |  |  |  |  |  |  |  |

| *Table S15A: Random effects of Maternal Hardship at Birth on SMFQ Trajectories* | | | | | | | | |
| --- | --- | --- | --- | --- | --- | --- | --- | --- |
|  | Unadjusted Model | | | | Adjusted Model | | | |
| Parameter | Estimate | Std. Error | Low 95% CIs | High 95% CIs | Estimate | Std. Error | Low 95% CIs | High 95% CIs |
| var(Intercept) | 18.70029 | 0.477277 | 17.76484 | 19.63573 | 17.3621 | 0.470235 | 16.44045 | 18.28374 |
| cov(Intercept, Age) | 0.43786 | 0.085557 | 0.2701714 | 0.6055492 | 0.437504 | 0.08571 | 0.2695144 | 0.6054932 |
| var(Age) | 0.397149 | 0.028852 | 0.3405998 | 0.4536983 | 0.391352 | 0.029489 | 0.3335555 | 0.4491492 |
| cov(Intercept, Age^2) | -0.50114 | 0.026261 | -0.5526098 | -0.4496696 | -0.46538 | 0.026231 | -0.5167887 | -0.4139652 |
| cov(Age, Age^2) | 0.022682 | 0.005558 | 0.0117887 | 0.0335749 | 0.022461 | 0.005667 | 0.0113534 | 0.0335688 |
| var(Age^2) | 0.027498 | 0.00217 | 0.0232445 | 0.0317508 | 0.02651 | 0.002208 | 0.0221835 | 0.0308373 |
| cov(Intercept, Age^3) | 0.008419 | 0.002492 | 0.0035345 | 0.0133043 | 0.006971 | 0.002494 | 0.0020828 | 0.0118598 |
| cov(Age, Age^3) | -0.00725 | 0.00078 | -0.0087752 | -0.0057179 | -0.00716 | 0.000797 | -0.00872 | -0.0055943 |
| cov(Age^2, Age^3) | -0.00075 | 0.000168 | -0.0010776 | -0.000421 | -0.00073 | 0.000171 | -0.0010654 | -0.000396 |
| var(Age^3) | 0.00019 | 2.26E-05 | 0.0001455 | 0.0002342 | 0.000186 | 2.31E-05 | 0.0001408 | 0.0002315 |
| cov(Intercept, Age^4) | 0.006549 | 0.000509 | 0.005552 | 0.0075459 | 0.005981 | 0.000508 | 0.0049849 | 0.0069774 |
| cov(Age, Age^4) | -0.00061 | 0.000117 | -0.0008377 | -0.0003788 | -0.0006 | 1.20E-04 | -0.0008388 | -0.0003699 |
| cov(Age^2, Age^4) | -0.00037 | 4.29E-05 | -4.58E-04 | -0.0002902 | -3.60E-04 | 4.37E-05 | -4.46E-04 | -2.75E-04 |
| cov(Age^3, Age^4) | 2.03E-05 | 3.52E-06 | 1.34E-05 | 2.72E-05 | 1.98E-05 | 3.60E-06 | 1.27E-05 | 2.68E-05 |
| var(Age^4) | 5.43E-06 | 8.98E-07 | 3.67E-06 | 7.19E-06 | 5.23E-06 | 9.16E-07 | 3.43E-06 | 7.03E-06 |

*Table S16: Maternal hardship comparisons at various ages of each trajectory*

|  | Age 10 | Age 14 | Age 18 | Age 22 |
| --- | --- | --- | --- | --- |
| Predicted low maternal hardship score | 3.71 (3.21, 4.21) | 4.12 (3.63, 4.61) | 5.70 (5.20, 6.20) | 5.13 (4.62, 5.63) |
| Predicted high maternal hardship score | 4.51 (3.95, 5.06) | 4.63 (4.12, 5.14) | 6.53 (5.98, 7.08) | 6.09 (5.50, 6.68) |
| Difference, *P*-Value | 0.79 (0.46, 1.13), = <.001 | 0.51 (0.28, 0.75), =<.001 | 0.83 (0.5, 1.15), <.001 | 0.96 (0.56, 1.36), <.001 |
| SMFQ % Difference | 3.04 | 1.96 | 3.19 | 3.69 |

*Adjusted for child sex, maternal age at birth, drinking in pregnancy, smoking in pregnancy, postnatal depression and intimate partner violence

Values in parentheses represent 95% confidence intervals

| *Table S17 Main Effects of Paternal Hardship (PHA) on SMFQ Trajectories* | | | | | | | | | | |
| --- | --- | --- | --- | --- | --- | --- | --- | --- | --- | --- |
|  | Unadjusted Model (n=5417) | | | | | Adjusted Model (n=3734) | | | | |
| Parameter | Estimate | Low 95% CIs | High 95% CIs | Std. Error | *p*-value | Estimate | Low 95% CIs | High 95% CIs | Std. Error | *p*-value |
| *β*0 - No PHA Intercept (ref) | 6.204 | 6.034 | 6.374 | 0.087 | <.001 | 5.154 | 4.672 | 5.635 | 0.246 | <.001 |
| *β*1 - No PHAxAge (ref) | 0.115 | 0.071 | 0.160 | 0.023 | <.001 | 0.122 | 0.071 | 0.173 | 0.026 | <.001 |
| *β*2 - No PHAxAge^2 (ref) | -0.091 | -0.102 | -0.079 | 0.006 | <.001 | -0.089 | -0.102 | -0.076 | 0.007 | <.001 |
| *β*3 - No PHAxAge^3 (ref) | 0.003 | 0.002 | 0.004 | 0.001 | <.001 | 0.002 | 0.001 | 0.004 | 0.001 | 0.001 |
| *β*4 - No PHAxAge^4 (ref) | 0.002 | 0.001 | 0.002 | 0.0001 | <.001 | 0.002 | 0.001 | 0.002 | 0.0001 | <.001 |
| *β*5 - PHA Intercept | 0.922 | 0.577 | 1.266 | 0.176 | <.001 | 1.066 | 0.649 | 1.483 | 0.213 | <.001 |
| *β*6 - PHAxAge | -0.019 | -0.111 | 0.072 | 0.047 | 0.676 | -0.071 | -0.181 | 0.039 | 0.056 | 0.205 |
| *β*7 - PHAxAge^2 | -0.027 | -0.051 | -0.003 | 0.012 | 0.027 | -0.039 | -0.068 | -0.011 | 0.015 | 0.007 |
| *β*8 - PHAxAge^3 | 0.002 | -0.001 | 0.004 | 0.001 | 0.214 | 0.003 | 0.0003 | 0.007 | 0.002 | 0.031 |
| *β*9 - PHAxAge^4 | 0.001 | 0.0000 | 0.001 | 0.0003 | 0.041 | 0.001 | 0.0002 | 0.001 | 0.0003 | 0.009 |
| Deviance | 159001.3 | | | | | 111574.5 | | | | |
| *Adjusted for child sex, paternal age at birth, drinking in pregnancy, smoking in pregnancy, postnatal depression and physical abuse | | | | | | | | |  |  |
| *Ref is the reference category which should be added to each category  *Intercepts are at age 18 to coincide with the end of adolescence | | | |  |  |  |  |  |  |  |

| *Table S17A: Random effects of Paternal Hardship at Birth on SMFQ Trajectories* | | | | | | | | |
| --- | --- | --- | --- | --- | --- | --- | --- | --- |
|  | Unadjusted Model | | | | Adjusted Model | | | |
| Parameter | Estimate | Std. Error | Low 95% CIs | High 95% CIs | Estimate | Std. Error | Low 95% CIs | High 95% CIs |
| var(Intercept) | 17.82904 | 0.544365 | 16.7621 | 18.89597 | 16.73566 | 0.61018 | 15.53973 | 17.93159 |
| cov(Intercept, Age) | 0.480965 | 0.09864 | 0.2876344 | 0.6742954 | 0.541435 | 0.111287 | 0.323317 | 0.7595535 |
| var(Age) | 0.425271 | 0.033828 | 0.3589695 | 0.4915731 | 0.423055 | 0.038591 | 0.3474189 | 0.4986912 |
| cov(Intercept, Age^2) | -0.48135 | 0.029936 | -0.5400224 | -0.4226752 | -0.46028 | 0.033574 | -0.5260807 | -0.3944751 |
| cov(Age, Age^2) | 0.020797 | 0.006412 | 0.0082294 | 0.0333636 | 0.015739 | 0.007212 | 0.0016042 | 0.0298734 |
| var(Age^2) | 0.026558 | 0.002474 | 0.0217088 | 0.0314064 | 0.024552 | 0.002764 | 0.0191342 | 0.0299698 |
| cov(Intercept, Age^3) | 0.006939 | 0.002841 | 0.0013702 | 0.012507 | 0.003947 | 0.003214 | -0.0023529 | 0.0102475 |
| cov(Age, Age^3) | -0.00791 | 0.000908 | -0.0096887 | -0.0061296 | -0.00799 | 0.001041 | -0.0100327 | -0.0059519 |
| cov(Age^2, Age^3) | -0.00065 | 0.000191 | -0.001027 | -0.0002785 | -0.00056 | 0.000216 | -0.0009878 | -0.0001402 |
| var(Age^3) | 0.0002 | 2.61E-05 | 0.0001489 | 0.0002512 | 0.000204 | 3.01E-05 | 0.0001451 | 0.0002631 |
| cov(Intercept, Age^4) | 0.006283 | 0.000576 | 0.0051541 | 0.0074125 | 0.005833 | 0.000645 | 0.0045683 | 0.0070968 |
| cov(Age, Age^4) | -0.0006 | 0.000135 | -0.0008615 | -0.0003341 | -0.00052 | 0.000152 | -0.0008145 | -0.0002185 |
| cov(Age^2, Age^4) | -0.00035 | 4.87E-05 | -4.50E-04 | -0.0002594 | -0.00032 | 5.44E-05 | -0.0004239 | -0.0002105 |
| cov(Age^3, Age^4) | 1.84E-05 | 4.00E-06 | 1.06E-05 | 2.63E-05 | 1.73E-05 | 4.56E-06 | 8.40E-06 | 2.63E-05 |
| var(Age^4) | 4.89E-06 | 1.01E-06 | 2.90E-06 | 6.87E-06 | 4.26E-06 | 1.14E-06 | 2.03E-06 | 6.49E-06 |

*Table S18: Paternal hardship comparisons at various ages of each trajectory*

|  | Age 10 | Age 14 | Age 18 | Age 22 |
| --- | --- | --- | --- | --- |
| Predicted low hardship paternal score | 3.21 (2.73, 3.70) | 3.58 (3.12, 4.04) | 5.17 (4.69, 5.66) | 4.75 (4.26, 5.25) |
| Predicted high hardship paternal score | 3.72 (3.14, 4.30) | 4.32 (3.80, 4.83) | 6.22 (5.65, 6.80) | 5.33 (4.71, 5.96) |
| Difference, *P*-Value | 0.50 (0.08, 0.93), =.021 | 0.74 (0.44, 1.05), <.001 | 1.05 (0.63, 1.57), <.001 | 0.58 (0.08, 1.08), =.023 |
| SMFQ % Difference | 1.92 | 2.84 | 4.04 | 2.23 |

*Adjusted for child sex, maternal age at birth, drinking in pregnancy, smoking in pregnancy, postnatal depression and intimate partner violence

Values in parentheses represent 95% confidence intervals

| *Table S19: Maternal Personality x Education x Time on SMFQ Trajectories* | | | | | | | | | | |
| --- | --- | --- | --- | --- | --- | --- | --- | --- | --- | --- |
|  | Unadjusted Model (n=6612) | | | | | Adjusted Model (n=6152) | | | | |
| Parameter | Estimate | Low 95% CIs | High 95% CIs | Std. Error | *p*-value | Estimate | Low 95% CIs | High 95% CIs | Std. Error | *p*-value |
| *β*0 - No PersonaliltyxHigher Education Intercept (ref) | 6.033 | 5.829 | 6.237 | 0.1 | <.001 | 5.412 | 4.827 | 5.997 | 0.3 | <.001 |
| *β*1 - No PersonaliltyxHigher EducationxAge (ref) | 0.06 | 0.008 | 0.112 | 0.03 | 0.024 | 0.052 | -0.001 | 0.105 | 0.03 | 0.053 |
| *β*2 - No PersonaliltyxHigher EducationxAge^2 (ref) | -0.079 | -0.093 | -0.065 | 0.01 | <.001 | -0.08 | -0.094 | -0.066 | 0.01 | <.001 |
| *β*3 - No PersonaliltyxHigher EducationxAge^3 (ref) | 0.004 | 0.002 | 0.006 | 0.001 | <.001 | 0.004 | 0.003 | 0.006 | 0.001 | <.001 |
| *β*4 - No PersonaliltyxHigher EducationxAge^4 (ref) | 0.001 | 0.001 | 0.002 | 0.0001 | <.001 | 0.001 | 0.001 | 0.002 | 0.0001 | <.001 |
| *β*5 - PersonalityxLower Education Intercept | 2.124 | 1.613 | 2.635 | 0.26 | <.001 | 1.611 | 1.076 | 2.145 | 0.27 | <.001 |
| *β*6 - PersonalityxLower EducationxAge | 0.227 | 0.089 | 0.365 | 0.07 | 0.001 | 0.233 | 0.089 | 0.377 | 0.07 | 0.002 |
| *β*7 - PersonalityxLower EducationxAge^2 | -0.066 | -0.102 | -0.03 | 0.02 | <.001 | -0.055 | -0.092 | -0.017 | 0.02 | 0.004 |
| *β*8 - PersonalityxLower EducationxAge^3 | -0.004 | -0.008 | 0.0002 | 0.002 | 0.061 | -0.005 | -0.009 | -0.0005 | 0.002 | 0.03 |
| *β*9 - PersonalityxLower EducationxAge^4 | 0.001 | 0.0001 | 0.002 | 0.0004 | 0.02 | 0.001 | -0.0002 | 0.001 | 0.0004 | 0.12 |
| *β*10 - PersonalityxHigher Education Intercept | 0.76 | 0.146 | 1.373 | 0.31 | 0.02 | 0.362 | -0.262 | 0.985 | 0.32 | 0.26 |
| *β*11 - PersonalityxHigher EducationxAge | 0.004 | -0.153 | 0.162 | 0.08 | 0.96 | 0.047 | -0.115 | 0.209 | 0.08 | 0.57 |
| *β*12 - PersonalityxHigher EducationxAge^2 | -0.016 | -0.058 | 0.026 | 0.02 | 0.46 | -0.004 | -0.047 | 0.039 | 0.02 | 0.85 |
| *β*13 - PersonalityxHigher EducationxAge^3 | 0.002 | -0.003 | 0.006 | 0.002 | 0.5 | 0.0001 | -0.005 | 0.005 | 0.002 | 0.98 |
| *β*14 - PersonalityxHigher EducationxAge^4 | 0.0004 | -0.0004 | 0.001 | 0.0004 | 0.325 | 0.0002 | -0.001 | 0.001 | 0.0005 | 0.68 |
| *β*15 - No PersonaliltyxLower Education Intercept | 0.438 | 0.148 | 0.728 | 0.15 | 0.003 | 0.364 | 0.067 | 0.66 | 0.15 | 0.02 |
| *β*16 - No PersonaliltyxLower EducationxAge | 0.045 | -0.03 | 0.121 | 0.04 | 0.239 | 0.037 | -0.04 | 0.114 | 0.04 | 0.35 |
| *β*17 - No PersonaliltyxLower EducationxAge^2 | -0.031 | -0.051 | -0.012 | 0.01 | 0.002 | -0.031 | -0.051 | -0.011 | 0.01 | 0.003 |
| *β*18 - No PersonaliltyxLower EducationxAge^3 | -0.0001 | -0.002 | 0.002 | 0.001 | 0.937 | 0.00001 | -0.002 | 0.002 | 0.001 | 0.99 |
| *β*19 - No PersonaliltyxLower EducationxAge^4 | 0.001 | 0.0002 | 0.001 | 0.0002 | 0.005 | 0.001 | 0.0002 | 0.001 | 0.0002 | 0.01 |
| Deviance | 196557.37 | | | | | 184334.87 | | | | |
| *Adjusted for child sex, maternal age at birth, drinking in pregnancy, smoking in pregnancy, postnatal depression and physical abuse | | | | | | | | | | |
| *Ref is the reference category which should be added to each category. *Intercepts are at age 18 to coincide with the end of adolescence | | | | | | | | | | |

| *Table S19A: Random effects of Maternal Personality x Education x Time on SMFQ Trajectories* | | | | | | | | |
| --- | --- | --- | --- | --- | --- | --- | --- | --- |
|  | Unadjusted Model | | | | Adjusted Model | | | |
| Parameter | Estimate | Std. Error | Low 95% CIs | High 95% CIs | Estimate | Std. Error | Low 95% CIs | High 95% CIs |
| var(Intercept) | 17.99877 | 0.496567 | 17.02551 | 18.97202 | 17.0344 | 0.49225 | 16.06961 | 17.99919 |
| cov(Intercept, Age) | 0.441895 | 0.089051 | 0.2673581 | 0.616431 | 0.462731 | 0.089193 | 0.2879155 | 0.6375467 |
| var(Age) | 0.38593 | 0.030051 | 0.3270303 | 0.4448295 | 0.374769 | 0.030483 | 0.3150237 | 0.434515 |
| cov(Intercept, Age^2) | -0.48056 | 0.027216 | -0.5338973 | -0.4272146 | -0.45747 | 0.027239 | -0.5108602 | -0.4040863 |
| cov(Age, Age^2) | 0.024881 | 0.005817 | 0.0134792 | 0.0362829 | 0.021826 | 0.005872 | 0.0103166 | 0.0333351 |
| var(Age^2) | 0.027347 | 0.002254 | 0.0229306 | 0.031764 | 0.025856 | 0.002277 | 0.0213926 | 0.0303183 |
| cov(Intercept, Age^3) | 0.007911 | 0.002608 | 0.0027989 | 0.0130227 | 0.006437 | 0.002608 | 0.0013249 | 0.0115486 |
| cov(Age, Age^3) | -0.00699 | 0.000816 | -0.0085899 | -0.0053904 | -0.00668 | 0.000827 | -0.0083049 | -0.0050637 |
| cov(Age^2, Age^3) | -0.00081 | 0.000176 | -0.0011577 | -0.0004668 | -0.00072 | 0.000178 | -0.0010673 | -0.0003705 |
| var(Age^3) | 0.000183 | 2.38E-05 | 0.0001367 | 0.00023 | 0.000174 | 2.41E-05 | 0.0001263 | 0.0002207 |
| cov(Intercept, Age^4) | 0.006246 | 0.00053 | 0.0052073 | 0.0072842 | 0.005846 | 0.000529 | 0.0048098 | 0.0068831 |
| cov(Age, Age^4) | -0.00066 | 0.000124 | -0.0009041 | -0.000419 | -0.00059 | 0.000125 | -0.0008345 | -0.0003458 |
| cov(Age^2, Age^4) | -0.00038 | 4.48E-05 | -0.0004691 | -0.0002935 | -0.00035 | 4.52E-05 | -0.0004392 | -0.0002621 |
| cov(Age^3, Age^4) | 2.18E-05 | 3.75E-06 | 0.0000144 | 0.0000291 | 1.94E-05 | 3.77E-06 | 0.000012 | 0.0000268 |
| var(Age^4) | 5.73E-06 | 9.44E-07 | 3.88E-06 | 7.58E-06 | 5.08E-06 | 9.51E-07 | 3.22E-06 | 6.94E-06 |

| *Table S20: Maternal Personality x Maternal Hardship (MHA) x Time on SMFQ Trajectories* | | | | | | | | | | |
| --- | --- | --- | --- | --- | --- | --- | --- | --- | --- | --- |
|  | Unadjusted Model (n=6446) | | | | | Adjusted Model (n=6021) | | | | |
| Parameter | Estimate | Low 95% CIs | High 95% CIs | Std. Error | *p*-value | Estimate | Low 95% CIs | High 95% CIs | Std. Error | *p*-value |
| *β*0 - No PersonaliltyxNo MHA Intercept (ref) | 6.113 | 5.951 | 6.275 | 0.083 | <.001 | 5.389 | 4.844 | 5.934 | 0.278 | <.001 |
| *β*1 - No PersonaliltyxNo MHAxAge (ref) | 0.073 | 0.032 | 0.115 | 0.021 | 0.001 | 0.063 | 0.021 | 0.105 | 0.022 | 0.003 |
| *β*2 - No PersonaliltyxNo MHAxAge^2 (ref) | -0.095 | -0.106 | -0.084 | 0.006 | <.001 | -0.095 | -0.106 | -0.084 | 0.006 | <.001 |
| *β*3 - No PersonaliltyxNo MHAxAge^3 (ref) | 0.004 | 0.003 | 0.005 | 0.001 | <.001 | 0.004 | 0.003 | 0.005 | 0.001 | <.001 |
| *β*4 - No PersonaliltyxNo MHAxAge^4 (ref) | 0.002 | 0.001 | 0.002 | 0.0001 | <.001 | 0.002 | 0.001 | 0.002 | 0.0001 | <.001 |
| *β*5 - PersonalityxNo MHA Intercept | 1.135 | 0.657 | 1.614 | 0.244 | <.001 | 0.747 | 0.258 | 1.237 | 0.25 | 0.003 |
| *β*6 - PersonalityxNo MHAxAge | 0.053 | -0.072 | 0.177 | 0.064 | 0.405 | 0.087 | -0.042 | 0.217 | 0.066 | 0.186 |
| *β*7 - PersonalityxNo MHAxAge^2 | -0.036 | -0.069 | -0.003 | 0.017 | 0.033 | -0.021 | -0.055 | 0.013 | 0.017 | 0.235 |
| *β*8 - PersonalityxNo MHAxAge^3 | 0.0001 | -0.004 | 0.004 | 0.002 | 0.96 | -0.001 | -0.005 | 0.003 | 0.002 | 0.52 |
| *β*9 - PersonalityxNo MHAxAge^4 | 0.001 | 0 | 0.001 | 0.0004 | 0.067 | 0.0003 | -0.0004 | 0.001 | 0.0004 | 0.358 |
| *β*10 - PersonalityxYes MHA Intercept | 2.05 | 1.391 | 2.71 | 0.337 | <.001 | 1.546 | 0.854 | 2.237 | 0.353 | <.001 |
| *β*11 - PersonalityxYes MHAxAge | 0.259 | 0.079 | 0.438 | 0.091 | 0.005 | 0.264 | 0.078 | 0.45 | 0.095 | 0.005 |
| β12 - PersonalityxYes MHAxAge^2 | -0.019 | -0.065 | 0.027 | 0.024 | 0.423 | -0.012 | -0.06 | 0.036 | 0.025 | 0.625 |
| β13 - PersonalityxYes MHAxAge^3 | -0.006 | -0.011 | -0.0003 | 0.003 | 0.036 | -0.006 | -0.011 | -0.001 | 0.003 | 0.03 |
| β14 - PersonalityxYes MHAxAge^4 | 0 | -0.001 | 0.001 | 0.0005 | 0.992 | -0.0001 | -0.001 | 0.001 | 0.001 | 0.799 |
| β15 - No PersonaliltyxYes MHA Intercept | 0.813 | 0.428 | 1.198 | 0.196 | <.001 | 0.723 | 0.326 | 1.119 | 0.202 | <.001 |
| β16 - No PersonaliltyxYes MHAxAge | 0.044 | -0.058 | 0.146 | 0.052 | 0.399 | 0.043 | -0.063 | 0.149 | 0.054 | 0.423 |
| β17 - No PersonaliltyxYes MHAxAge^2 | -0.003 | -0.03 | 0.023 | 0.014 | 0.798 | -0.007 | -0.034 | 0.021 | 0.014 | 0.634 |
| β18 - No PersonaliltyxYes MHAxAge^3 | -0.0001 | -0.003 | 0.003 | 0.002 | 0.926 | 0.0001 | -0.003 | 0.003 | 0.002 | 0.933 |
| β19 - No PersonaliltyxYes MHAxAge^4 | 0.0002 | -0.0004 | 0.001 | 0.0003 | 0.588 | 0.0002 | -0.0003 | 0.001 | 0.0003 | 0.42 |
| Deviance | 191730.33 | | | | | 180619.72 | | | | |
| *Adjusted for child sex, maternal age at birth, drinking in pregnancy, smoking in pregnancy, postnatal depression and physical abuse | | | | | | | |  |  |  |
| *Ref is the reference category which should be added to each category | | | |  |  |  |  |  |  |  |
| *Intercepts are at age 18 to coincide with the end of adolescence | |  |  |  |  |  |  |  |  |  |

| *Table S20A: Random effects of Maternal Personality x Maternal Hardship x Time on SMFQ Trajectories* | | | | | | | | |
| --- | --- | --- | --- | --- | --- | --- | --- | --- |
|  | Unadjusted Model | | | | Adjusted Model | | | |
| Parameter | Estimate | Std. Error | Low 95% CIs | High 95% CIs | Estimate | Std. Error | Low 95% CIs | High 95% CIs |
| var(Intercept) | 17.95299 | 0.502746 | 16.96763 | 18.93836 | 17.06837 | 0.499012 | 16.09032 | 18.04641 |
| cov(Intercept, Age) | 0.456456 | 0.089771 | 0.2805086 | 0.632403 | 0.472634 | 0.090065 | 0.2961101 | 0.6491576 |
| var(Age) | 0.379926 | 0.030229 | 0.3206792 | 0.4391735 | 0.371639 | 0.030699 | 0.3114697 | 0.4318089 |
| cov(Intercept, Age^2) | -0.4829 | 0.027499 | -0.5367949 | -0.4290015 | -0.4615 | 0.027618 | -0.5156357 | -0.4073736 |
| cov(Age, Age^2) | 0.021027 | 0.005822 | 0.0096164 | 0.0324372 | 0.020444 | 0.005918 | 0.0088456 | 0.0320419 |
| var(Age^2) | 0.02645 | 0.002263 | 0.0220145 | 0.0308856 | 0.025893 | 0.002304 | 0.0213763 | 0.0304089 |
| cov(Intercept, Age^3) | 0.006715 | 0.002624 | 0.0015727 | 0.0118579 | 0.005667 | 0.00263 | 0.0005132 | 0.0108215 |
| cov(Age, Age^3) | -0.00683 | 0.000819 | -0.0084383 | -0.0052262 | -0.00663 | 0.000832 | -0.0082593 | -0.0049983 |
| cov(Age^2, Age^3) | -0.00068 | 0.000176 | -0.0010201 | -0.0003308 | -0.00066 | 0.000179 | -0.0010128 | -0.000312 |
| var(Age^3) | 0.000177 | 2.38E-05 | 0.0001305 | 0.000224 | 0.000171 | 2.42E-05 | 0.0001238 | 0.0002187 |
| cov(Intercept, Age^4) | 0.006178 | 0.000532 | 0.0051352 | 0.0072213 | 0.005844 | 0.000534 | 0.0047969 | 0.006891 |
| cov(Age, Age^4) | -0.00058 | 0.000123 | -0.0008262 | -0.0003433 | -0.00057 | 0.000125 | -0.0008104 | -0.0003196 |
| cov(Age^2, Age^4) | -0.00036 | 4.48E-05 | -0.0004429 | -0.0002674 | -0.00035 | 4.56E-05 | -0.000436 | -0.0002574 |
| cov(Age^3, Age^4) | 1.91E-05 | 3.72E-06 | 0.0000118 | 0.0000264 | 1.84E-05 | 3.78E-06 | 0.000011 | 0.0000259 |
| var(Age^4) | 5.10E-06 | 9.39E-07 | 3.26E-06 | 6.94E-06 | 4.95E-06 | 9.57E-07 | 3.08E-06 | 6.83E-06 |

| *Table S21: Main effects of Paternal Personality x Education x Time on SMFQ Trajectories* | | | | | | | | | | |
| --- | --- | --- | --- | --- | --- | --- | --- | --- | --- | --- |
|  | Unadjusted Model (n=2741) | | | | | Adjusted Model (n=2009) | | | | |
| Parameter | Estimate | Low 95% CIs | High 95% CIs | Std. Error | *p*-value | Estimate | Low 95% CIs | High 95% CIs | Std. Error | *p*-value |
| *β*0 - No PersonaliltyxHigher Education Intercept (ref) | 6.249 | 5.996 | 6.502 | 0.129 | <.001 | 5.630 | 4.981 | 6.279 | 0.331 | <.001 |
| *β*1 - No PersonaliltyxHigher EducationxAge (ref) | 0.089 | 0.026 | 0.153 | 0.033 | 0.006 | 0.096 | 0.025 | 0.167 | 0.036 | 0.008 |
| *β*2 - No PersonaliltyxHigher EducationxAge^2 (ref) | -0.086 | -0.102 | -0.069 | 0.008 | <.001 | -0.084 | -0.102 | -0.066 | 0.009 | <.001 |
| *β*3 - No PersonaliltyxHigher EducationxAge^3 (ref) | 0.004 | 0.002 | 0.006 | 0.001 | <.001 | 0.003 | 0.001 | 0.005 | 0.001 | 0.002 |
| *β*4 - No PersonaliltyxHigher EducationxAge^4 (ref) | 0.001 | 0.001 | 0.002 | 0.0002 | <.001 | 0.001 | 0.001 | 0.002 | 0.000 | <.001 |
| *β*5 - PersonalityxLower Education Intercept | 0.795 | -0.193 | 1.783 | 0.504 | 0.115 | 1.184 | -0.088 | 2.456 | 0.649 | 0.068 |
| *β*6 - PersonalityxLower EducationxAge | -0.180 | -0.433 | 0.072 | 0.129 | 0.162 | -0.261 | -0.589 | 0.067 | 0.167 | 0.119 |
| *β*7 - PersonalityxLower EducationxAge^2 | -0.076 | -0.143 | -0.009 | 0.034 | 0.027 | -0.096 | -0.179 | -0.012 | 0.043 | 0.025 |
| *β*8 - PersonalityxLower EducationxAge^3 | 0.003 | -0.004 | 0.011 | 0.004 | 0.375 | 0.006 | -0.003 | 0.016 | 0.005 | 0.203 |
| *β*9 - PersonalityxLower EducationxAge^4 | 0.001 | -0.0001 | 0.003 | 0.001 | 0.079 | 0.002 | -0.0002 | 0.003 | 0.001 | 0.085 |
| *β*10 - PersonalityxHigher Education Intercept | 0.159 | -0.755 | 1.072 | 0.466 | 0.734 | 0.162 | -0.893 | 1.217 | 0.538 | 0.763 |
| *β*11 - PersonalityxHigher EducationxAge | -0.052 | -0.276 | 0.172 | 0.114 | 0.650 | -0.081 | -0.334 | 0.173 | 0.129 | 0.532 |
| *β*12 - PersonalityxHigher EducationxAge^2 | 0.028 | -0.032 | 0.088 | 0.031 | 0.362 | 0.004 | -0.063 | 0.072 | 0.034 | 0.902 |
| *β*13 - PersonalityxHigher EducationxAge^3 | 0.002 | -0.005 | 0.008 | 0.003 | 0.635 | 0.003 | -0.005 | 0.010 | 0.004 | 0.440 |
| *β*14 - PersonalityxHigher EducationxAge^4 | -0.0004 | -0.002 | 0.001 | 0.001 | 0.531 | 0.0002 | -0.001 | 0.002 | 0.001 | 0.769 |
| *β*15 - No PersonaliltyxLower Education Intercept | -0.212 | -0.642 | 0.218 | 0.219 | 0.334 | -0.241 | -0.738 | 0.256 | 0.253 | 0.342 |
| *β*16 - No PersonaliltyxLower EducationxAge | 0.022 | -0.088 | 0.133 | 0.056 | 0.694 | -0.015 | -0.142 | 0.112 | 0.065 | 0.811 |
| *β*17 - No PersonaliltyxLower EducationxAge^2 | -0.003 | -0.031 | 0.026 | 0.015 | 0.854 | -0.012 | -0.045 | 0.020 | 0.017 | 0.464 |
| *β*18 - No PersonaliltyxLower EducationxAge^3 | -0.001 | -0.004 | 0.003 | 0.002 | 0.713 | 0.0004 | -0.003 | 0.004 | 0.002 | 0.822 |
| *β*19 - No PersonaliltyxLower EducationxAge^4 | 0.0001 | -0.0005 | 0.001 | 0.0003 | 0.674 | 0.0004 | -0.0003 | 0.001 | 0.0003 | 0.266 |
| Deviance | 89958.99 | | | | | 66567.2 | | | | |
| *Adjusted for child sex, paternal age at birth, drinking in pregnancy, smoking in pregnancy, postnatal depression and physical abuse | | | | | | | |  |  |  |
| *Ref is the reference category which should be added to each category | | |  |  |  |  |  |  |  |  |
| *Intercepts are at age 18 to coincide with the end of adolescence |  |  |  |  |  |  |  |  |  |  |

| *Table S21A: Random effects of Paternal Personality x Education x Time on SMFQ Trajectories* | | | | | | | | |
| --- | --- | --- | --- | --- | --- | --- | --- | --- |
|  | Unadjusted Model | | | | Adjusted Model | | | |
| Parameter | Estimate | Std. Error | Low 95% CIs | High 95% CIs | Estimate | Std. Error | Low 95% CIs | High 95% CIs |
| var(Intercept) | 17.30943 | 0.691283 | 15.95454 | 18.66432 | 16.679 | 0.770851 | 15.16816 | 18.18983 |
| cov(Intercept, Age) | 0.392525 | 0.121896 | 0.1536118 | 0.6314371 | 0.52175 | 0.136143 | 0.2549147 | 0.7885851 |
| var(Age) | 0.411033 | 0.041298 | 0.3300903 | 0.4919749 | 0.398071 | 0.045965 | 0.3079806 | 0.4881606 |
| cov(Intercept, Age^2) | -0.46783 | 0.036947 | -0.5402428 | -0.3954129 | -0.43781 | 0.040598 | -0.5173766 | -0.3582372 |
| cov(Age, Age^2) | 0.014858 | 0.007752 | -0.0003362 | 0.030052 | 0.011449 | 0.008497 | -0.0052053 | 0.0281029 |
| var(Age^2) | 0.025188 | 0.002969 | 0.0193676 | 0.0310075 | 0.022241 | 0.003225 | 0.01592 | 0.0285614 |
| cov(Intercept, Age^3) | 0.008329 | 0.003552 | 0.0013672 | 0.0152909 | 0.004162 | 0.004001 | -0.0036793 | 0.0120037 |
| cov(Age, Age^3) | -0.00774 | 0.001118 | -0.0099311 | -0.0055478 | -0.00773 | 0.001258 | -0.0101912 | -0.0052605 |
| cov(Age^2, Age^3) | -0.00059 | 0.000234 | -0.0010526 | -0.0001366 | -0.00047 | 0.000259 | -0.0009739 | 0.0000416 |
| var(Age^3) | 0.000205 | 3.26E-05 | 0.0001412 | 0.000269 | 0.000214 | 3.71E-05 | 0.0001414 | 0.0002869 |
| cov(Intercept, Age^4) | 0.006107 | 0.000714 | 0.0047072 | 0.007506 | 0.005404 | 0.000783 | 0.0038703 | 0.006938 |
| cov(Age, Age^4) | -0.00049 | 0.000165 | -0.0008142 | -0.0001682 | -0.00045 | 0.000181 | -0.0008021 | -0.0000929 |
| cov(Age^2, Age^4) | -0.00035 | 5.88E-05 | -0.0004611 | -0.0002305 | -0.00028 | 6.37E-05 | -0.0004042 | -0.0001546 |
| cov(Age^3, Age^4) | 1.84E-05 | 4.98E-06 | 8.69E-06 | 0.0000282 | 1.71E-05 | 5.53E-06 | 6.22E-06 | 0.0000279 |
| var(Age^4) | 5.10E-06 | 1.24E-06 | 2.68E-06 | 7.53E-06 | 3.79E-06 | 1.34E-06 | 1.16E-06 | 6.42E-06 |

| *Table S22: Paternal Personality x Paternal Hardship (PHA) x Time on SMFQ Trajectories* | | | | | | | | | | |
| --- | --- | --- | --- | --- | --- | --- | --- | --- | --- | --- |
|  | Unadjusted Model (n=2585) | | | | | Adjusted Model (n=1960) | | | | |
| Parameter | Estimate | Low 95% CIs | High 95% CIs | Std. Error | *p*-value | Estimate | Low 95% CIs | High 95% CIs | Std. Error | *p*-value |
| *β*0 - No PersonaliltyxNo PHA Intercept (ref) | 6.009 | 5.773 | 6.245 | 0.120 | <.001 | 5.362 | 4.721 | 6.004 | 0.327 | <.001 |
| *β*1 - No PersonaliltyxNo PHAxAge (ref) | 0.138 | 0.078 | 0.198 | 0.030 | <.001 | 0.137 | 0.071 | 0.203 | 0.034 | <.001 |
| *β*2 - No PersonaliltyxNo PHAxAge^2 (ref) | -0.082 | -0.098 | -0.067 | 0.008 | <.001 | -0.082 | -0.099 | -0.065 | 0.009 | <.001 |
| *β*3 - No PersonaliltyxNo PHAxAge^3 (ref) | 0.002 | 0.0003 | 0.004 | 0.001 | 0.021 | 0.002 | -0.0003 | 0.004 | 0.001 | 0.104 |
| *β*4 - No PersonaliltyxNo PHAxAge^4 (ref) | 0.001 | 0.001 | 0.002 | 0.0002 | <.001 | 0.001 | 0.001 | 0.002 | 0.0002 | <.001 |
| *β*5 - PersonalityxNo PHA Intercept | 0.444 | -0.439 | 1.328 | 0.451 | 0.324 | 0.129 | -0.871 | 1.129 | 0.510 | 0.801 |
| *β*6 - PersonalityxNo PHAxAge | -0.161 | -0.376 | 0.053 | 0.110 | 0.141 | -0.243 | -0.485 | -0.001 | 0.123 | 0.049 |
| *β*7 - PersonalityxNo PHAxAge^2 | -0.044 | -0.102 | 0.014 | 0.029 | 0.136 | -0.046 | -0.110 | 0.019 | 0.033 | 0.163 |
| *β*8 - PersonalityxNo PHAxAge^3 | 0.007 | 0.0003 | 0.013 | 0.003 | 0.041 | 0.010 | 0.003 | 0.018 | 0.004 | 0.005 |
| *β*9 - PersonalityxNo PHAxAge^4 | 0.001 | 0.0000 | 0.002 | 0.001 | 0.045 | 0.002 | 0.0003 | 0.003 | 0.001 | 0.019 |
| *β*10 - PersonalityxYes PHA Intercept | 1.434 | 0.319 | 2.549 | 0.569 | 0.012 | 2.096 | 0.687 | 3.505 | 0.719 | 0.004 |
| *β*11 - PersonalityxYes PHAxAge | 0.032 | -0.254 | 0.318 | 0.146 | 0.827 | 0.013 | -0.351 | 0.377 | 0.186 | 0.944 |
| β12 - PersonalityxYes PHAxAge^2 | -0.006 | -0.082 | 0.070 | 0.039 | 0.877 | -0.018 | -0.113 | 0.076 | 0.048 | 0.705 |
| β13 - PersonalityxYes PHAxAge^3 | -0.002 | -0.010 | 0.006 | 0.004 | 0.599 | -0.002 | -0.013 | 0.008 | 0.005 | 0.650 |
| β14 - PersonalityxYes PHAxAge^4 | -0.0005 | -0.002 | 0.001 | 0.001 | 0.555 | -0.001 | -0.002 | 0.001 | 0.001 | 0.601 |
| β15 - No PersonaliltyxYes PHA Intercept | 0.878 | 0.340 | 1.416 | 0.275 | 0.001 | 0.999 | 0.388 | 1.611 | 0.312 | 0.001 |
| β16 - No PersonaliltyxYes PHAxAge | -0.138 | -0.274 | -0.001 | 0.069 | 0.048 | -0.249 | -0.403 | -0.094 | 0.079 | 0.002 |
| β17 - No PersonaliltyxYes PHAxAge^2 | -0.031 | -0.067 | 0.004 | 0.018 | 0.082 | -0.052 | -0.092 | -0.012 | 0.020 | 0.011 |
| β18 - No PersonaliltyxYes PHAxAge^3 | 0.006 | 0.002 | 0.010 | 0.002 | 0.004 | 0.009 | 0.005 | 0.014 | 0.002 | <.001 |
| β19 - No PersonaliltyxYes PHAxAge^4 | 0.001 | 0.0001 | 0.002 | 0.0004 | 0.033 | 0.001 | 0.0005 | 0.002 | 0.0004 | 0.002 |
| Deviance | 85011.98 | | | | | 64925.72 | | | | |
| *Adjusted for child sex, paternal age at birth, drinking in pregnancy, smoking in pregnancy, postnatal depression and physical abuse | | | | | | | |  |  |  |
| *Ref is the reference category which should be added to each category | | | |  |  |  |  |  |  |  |
| *Intercepts are at age 18 to coincide with the end of adolescence | |  |  |  |  |  |  |  |  |  |

| *Table S22A: Random effects of Paternal Personality x Paternal Hardship x Time on SMFQ Trajectories* | | | | | | | | |
| --- | --- | --- | --- | --- | --- | --- | --- | --- |
|  | Unadjusted Model | | | | Adjusted Model | | | |
| Parameter | Estimate | Std. Error | Low 95% CIs | High 95% CIs | Estimate | Std. Error | Low 95% CIs | High 95% CIs |
| var(Intercept) | 17.65052 | 0.722369 | 16.23471 | 19.06634 | 16.84472 | 0.787555 | 15.30114 | 18.3883 |
| cov(Intercept, Age) | 0.483218 | 0.126665 | 0.2349586 | 0.731477 | 0.598271 | 0.138628 | 0.3265642 | 0.8699772 |
| var(Age) | 0.413822 | 0.042441 | 0.3306394 | 0.4970043 | 0.397762 | 0.04648 | 0.3066623 | 0.4888616 |
| cov(Intercept, Age^2) | -0.48769 | 0.038556 | -0.5632556 | -0.4121207 | -0.46121 | 0.041768 | -0.5430783 | -0.3793494 |
| cov(Age, Age^2) | 0.013885 | 0.007985 | -0.0017667 | 0.0295356 | 0.007031 | 0.008625 | -0.0098737 | 0.0239357 |
| var(Age^2) | 0.025905 | 0.003074 | 0.0198797 | 0.0319297 | 0.0234 | 0.003308 | 0.016916 | 0.0298845 |
| cov(Intercept, Age^3) | 0.005811 | 0.003669 | -0.001379 | 0.0130017 | 0.001382 | 0.004057 | -0.0065696 | 0.009333 |
| cov(Age, Age^3) | -0.00768 | 0.001143 | -0.0099166 | -0.0054348 | -0.00761 | 0.001268 | -0.0100896 | -0.0051204 |
| cov(Age^2, Age^3) | -0.00055 | 0.000239 | -0.0010173 | -0.000079 | -0.00036 | 0.000262 | -0.0008722 | 0.0001555 |
| var(Age^3) | 0.000201 | 3.32E-05 | 0.0001355 | 0.0002655 | 0.000208 | 3.73E-05 | 0.0001345 | 0.0002807 |
| cov(Intercept, Age^4) | 0.006348 | 0.00074 | 0.0048982 | 0.0077971 | 0.005675 | 0.000798 | 0.004111 | 0.0072393 |
| cov(Age, Age^4) | -0.00045 | 0.000168 | -0.0007802 | -0.0001213 | -0.00036 | 0.000182 | -0.0007132 | -4.37E-07 |
| cov(Age^2, Age^4) | -0.00035 | 6.05E-05 | -0.0004682 | -0.0002311 | -0.00029 | 6.49E-05 | -0.0004169 | -0.0001626 |
| cov(Age^3, Age^4) | 0.000017 | 5.05E-06 | 7.08E-06 | 0.0000269 | 1.49E-05 | 5.55E-06 | 4.03E-06 | 0.0000258 |
| var(Age^4) | 4.95E-06 | 1.26E-06 | 2.47E-06 | 7.43E-06 | 3.78E-06 | 1.36E-06 | 1.11E-06 | 6.44E-06 |

| *Table S23: Main Effects of Parental Personality on SMFQ Trajectories using Inverse Probability Weighting (IPW)* | | | | | | | | | | |
| --- | --- | --- | --- | --- | --- | --- | --- | --- | --- | --- |
|  | Maternal IPW Model (n=6232) | | | | | Paternal IPW Model (n=2098) | | | | |
| Parameter | Estimate | Low 95% CIs | High 95% CIs | Std. Error | *p*-value | Estimate | Low 95% CIs | High 95% CIs | Std. Error | *p*-value |
| *β*0 - No Parental Personality Intercept (ref) | 5.670 | 0.276 | 5.128 | 6.211 | <.001 | 5.431 | 0.318 | 4.808 | 6.053979 | <.001 |
| *β*1 - No Parental PersonalityxAge *(*ref*)* | 0.071 | 0.019 | 0.033 | 0.108 | <.001 | 0.085 | 0.030 | 0.026 | 0.14389 | 0.005 |
| *β*2 - No Parental PersonalityxAge^2 (ref) | -0.096 | 0.005 | -0.106 | -0.086 | <.001 | -0.092 | 0.008 | -0.107 | -0.07748 | <.001 |
| *β*3 - No Parental PersonalityAge^3 (ref) | 0.004 | 0.001 | 0.003 | 0.005 | <.001 | 0.004 | 0.001 | 0.002 | 0.00554 | <.001 |
| *β*4 - No Parental PersonalityxAge^4 (ref) | 0.002 | 0.0001 | 0.002 | 0.002 | <.001 | 0.002 | 0.0002 | 0.001 | 0.00194 | <.001 |
| *β*5 - Parental Personality Intercept | 0.951 | 0.233 | 0.495 | 1.407 | <.001 | 0.587 | 0.407 | -0.211 | 1.385365 | 0.149 |
| *β*6 - Parental PersonalityxAge | 0.137 | 0.062 | 0.015 | 0.258 | 0.028 | -0.140 | 0.100 | -0.336 | 0.055639 | 0.161 |
| *β*7 - Parental PersonalityxAge^2 | -0.021 | 0.016 | -0.053 | 0.010 | 0.178 | -0.032 | 0.029 | -0.089 | 0.02608 | 0.283 |
| *β*8 - Parental PersonalityxAge^3 | -0.003 | 0.002 | -0.006 | 0.001 | 0.144 | 0.004 | 0.003 | -0.002 | 0.009991 | 0.232 |
| *β*9 - Parental PersonalityxAge^4 | 0.0002 | 0.0003 | -0.0004 | 0.001 | 0.499 | 0.0006 | 0.0006 | -0.001 | 0.001797 | 0.308 |
| Deviance | 186596.9 | | | | | 69217.86 | | | | |
| *Adjusted for child sex, parental age at birth, drinking in pregnancy, smoking in pregnancy, postnatal depression and physical abuse | | | | | | | |  |  |  |
| *Ref is the reference category which should be added to each category | | | |  |  |  |  |  |  |  |
| *Intercepts are at age 18 to coincide with the end of adolescence | |  |  |  |  |  |  |  |  |  |

| *Table S23A: Random effects of Parental Personality on SMFQ Trajectories using Inverse Probability Weighting (IPW)* | | | | | | | | |
| --- | --- | --- | --- | --- | --- | --- | --- | --- |
|  | Maternal IPW Model (n=6232) | | | | Paternal IPW Model (n=2098) | | | |
| Parameter | Estimate | Std. Error | Low 95% CIs | High 95% CIs | Estimate | Std. Error | Low 95% CIs | High 95% CIs |
| var(Intercept) | 17.36019 | 0.59621 | 16.19164 | 18.52874 | 17.14669 | 0.977398 | 15.23103 | 19.06236 |
| cov(Intercept, Age) | 0.496823 | 0.105161 | 0.290711 | 0.702934 | 0.522813 | 0.171737 | 0.186214 | 0.859412 |
| var(Age) | 0.370412 | 0.036805 | 0.298276 | 0.442547 | 0.381802 | 0.055397 | 0.273226 | 0.490379 |
| cov(Intercept, Age^2) | -0.46719 | 0.032813 | -0.53151 | -0.40288 | -0.45882 | 0.050736 | -0.55827 | -0.35938 |
| cov(Age, Age^2) | 0.019812 | 0.007485 | 0.005142 | 0.034481 | 0.007936 | 0.0107 | -0.01304 | 0.028907 |
| var(Age^2) | 0.026109 | 0.002785 | 0.020651 | 0.031567 | 0.021995 | 0.003859 | 0.014432 | 0.029559 |
| cov(Intercept, Age^3) | 0.005877 | 0.00309 | -0.00018 | 0.011934 | 0.005127 | 0.004951 | -0.00458 | 0.01483 |
| cov(Age, Age^3) | -0.00658 | 0.000999 | -0.00853 | -0.00462 | -0.00734 | 0.001523 | -0.01033 | -0.00436 |
| cov(Age^2, Age^3) | -0.00067 | 0.000222 | -0.0011 | -0.00023 | -0.00042 | 0.000317 | -0.00104 | 0.000203 |
| var(Age^3) | 0.000172 | 0.000029 | 0.000115 | 0.000229 | 0.000208 | 4.48E-05 | 0.000121 | 0.000296 |
| cov(Intercept, Age^4) | 0.00597 | 0.000624 | 0.004748 | 0.007193 | 0.005779 | 0.000963 | 0.003891 | 0.007667 |
| cov(Age, Age^4) | -0.00056 | 0.000159 | -0.00087 | -0.00024 | -0.00036 | 0.000221 | -0.00079 | 7.24E-05 |
| cov(Age^2, Age^4) | -0.00035 | 5.44E-05 | -0.00046 | -0.00024 | -0.00027 | 7.36E-05 | -0.00041 | -0.00012 |
| cov(Age^3, Age^4) | 1.86E-05 | 4.72E-06 | 9.40E-06 | 2.79E-05 | 1.58E-05 | 6.55E-06 | 2.96E-06 | 2.86E-05 |
| var(Age^4) | 5.02E-06 | 1.14E-06 | 2.79E-06 | 7.25E-06 | 3.43E-06 | 1.52E-06 | 4.52E-07 | 6.40E-06 |

| *Table S24: Main Effects of Parental Education on SMFQ Trajectories using Inverse Probability Weighting (IPW)* | | | | | | | | | | |
| --- | --- | --- | --- | --- | --- | --- | --- | --- | --- | --- |
|  | Maternal IPW Model (n=7577) | | | | | Paternal IPW Model (n=4159) | | | | |
| Parameter | Estimate | Low 95% CIs | High 95% CIs | Std. Error | *p*-value | Estimate | Low 95% CIs | High 95% CIs | Std. Error | *p*-value |
| *β*0 - No Parental Education Intercept (ref) | 6.183 | 0.263 | 5.666 | 6.69866 | <.001 | 5.573 | 0.257 | 5.068999 | 6.076063 | <.001 |
| *β*1 - No Parental EducationxAge *(*ref*)* | 0.121 | 0.025 | 0.072 | 0.170067 | <.001 | 0.051 | 0.036 | -0.01958 | 0.122042 | 0.156 |
| *β*2 - No Parental EducationxAge^2 (ref) | -0.117 | 0.007 | -0.130 | -0.10352 | <.001 | -0.122 | 0.010 | -0.141 | -0.10265 | <.001 |
| *β*3 - No Parental EducationxAge^3 (ref) | 0.004 | 0.0007 | 0.002 | 0.005038 | <.001 | 0.005 | 0.0011 | 0.003022 | 0.007205 | <.001 |
| *β*4 - No Parental EducationxAge^4 (ref) | 0.002 | 0.000 | 0.002 | 0.002353 | <.001 | 0.002 | 0.000 | 0.001797 | 0.002596 | <.001 |
| *β*5 - Parental Education Intercept | -0.535 | 0.131 | -0.791 | -0.27835 | <.001 | -0.314 | 0.174 | -0.65571 | 0.027593 | 0.072 |
| *β*6 - Parental EducationxAge | -0.065 | 0.034 | -0.133 | 0.002066 | 0.057 | 0.055 | 0.045 | -0.03411 | 0.144129 | 0.226 |
| *β*7 - Parental EducationxAge^2 | 0.036 | 0.009 | 0.019 | 0.054101 | <.001 | 0.039 | 0.012 | 0.015669 | 0.062857 | 0.001 |
| *β*8 - Parental EducationxAge^3 | 0.0007 | 0.0010 | -0.001 | 0.002666 | 0.484 | -0.0020 | 0.0013 | -0.0046 | 0.000647 | 0.14 |
| *β*9 - Parental EducationxAge^4 | -6E-04 | 0.000189 | -0.00099 | -0.00026 | 0.001 | -8E-04 | 0.000252 | -0.00125 | -0.00027 | 0.003 |
| Deviance | 212848.39 | | | | | 123240.71 | | | | |
| *Adjusted for child sex, parental age at birth, drinking in pregnancy, smoking in pregnancy, postnatal depression and physical abuse | | | | | | | |  |  |  |
| *Ref is the reference category which should be added to each category | | | |  |  |  |  |  |  |  |
| *Intercepts are at age 18 to coincide with the end of adolescence | |  |  |  |  |  |  |  |  |  |

| *Table S24A: Random effects of Parental Education on SMFQ Trajectories using Inverse Probability Weighting (IPW)* | | | | | | | | |
| --- | --- | --- | --- | --- | --- | --- | --- | --- |
|  | Maternal IPW Model (n=7577) | | | | Paternal IPW Model (n=4159) | | | |
| Parameter | Estimate | Std. Error | Low 95% CIs | High 95% CIs | Estimate | Std. Error | Low 95% CIs | High 95% CIs |
| var(Intercept) | 17.4643 | 0.542597 | 16.40083 | 18.52777 | 16.73015 | 0.700846 | 15.35651 | 18.10378 |
| cov(Intercept, Age) | 0.434471 | 0.09944 | 0.239573 | 0.62937 | 0.412409 | 0.127814 | 0.161898 | 0.66292 |
| var(Age) | 0.394998 | 0.035467 | 0.325484 | 0.464512 | 0.422343 | 0.045085 | 0.333978 | 0.510708 |
| cov(Intercept, Age^2) | -0.46607 | 0.030318 | -0.52549 | -0.40665 | -0.45559 | 0.039068 | -0.53216 | -0.37902 |
| cov(Age, Age^2) | 0.023368 | 0.007159 | 0.009337 | 0.037399 | 0.023744 | 0.008939 | 0.006225 | 0.041263 |
| var(Age^2) | 0.026428 | 0.002652 | 0.02123 | 0.031626 | 0.02575 | 0.003271 | 0.01934 | 0.032161 |
| cov(Intercept, Age^3) | 0.007661 | 0.002891 | 0.001995 | 0.013327 | 0.007855 | 0.00369 | 0.000622 | 0.015087 |
| cov(Age, Age^3) | -0.0072 | 0.000961 | -0.00908 | -0.00532 | -0.00837 | 0.001248 | -0.01081 | -0.00592 |
| cov(Age^2, Age^3) | -0.00077 | 0.000211 | -0.00119 | -0.00036 | -0.00084 | 0.000267 | -0.00136 | -0.00032 |
| var(Age^3) | 0.000187 | 2.77E-05 | 0.000133 | 0.000242 | 0.000226 | 3.65E-05 | 0.000155 | 0.000298 |
| cov(Intercept, Age^4) | 0.006041 | 0.00058 | 0.004906 | 0.007177 | 0.005948 | 0.000743 | 0.004493 | 0.007404 |
| cov(Age, Age^4) | -0.00062 | 0.000152 | -0.00092 | -0.00032 | -0.0007 | 0.000191 | -0.00107 | -0.00032 |
| cov(Age^2, Age^4) | -0.00036 | 5.19E-05 | -0.00046 | -0.00026 | -0.00036 | 6.41E-05 | -0.00049 | -0.00024 |
| cov(Age^3, Age^4) | 2.05E-05 | 4.49E-06 | 1.17E-05 | 2.93E-05 | 2.39E-05 | 5.73E-06 | 1.27E-05 | 3.51E-05 |
| var(Age^4) | 5.27E-06 | 1.09E-06 | 3.14E-06 | 7.40E-06 | 5.62E-06 | 1.35E-06 | 2.98E-06 | 8.27E-06 |

| *Table S25: Main Effects of Parental Hardship on SMFQ Trajectories using Inverse Probability Weighting (IPW)* | | | | | | | | | | |
| --- | --- | --- | --- | --- | --- | --- | --- | --- | --- | --- |
|  | Maternal IPW Model (n=7412) | | | | | Paternal IPW Model (n=3734) | | | | |
| Parameter | Estimate | Low 95% CIs | High 95% CIs | Std. Error | *p*-value | Estimate | Low 95% CIs | High 95% CIs | Std. Error | *p*-value |
| *β*0 - No Parental Hardship Intercept (ref) | 5.704 | 0.260 | 5.193592 | 6.213565 | <.001 | 5.153 | 0.251 | 4.66188 | 5.64404 | <.001 |
| *β*1 - No Parental HardshipxAge *(*ref*)* | 0.076 | 0.019 | 0.038887 | 0.112294 | <.001 | 0.121 | 0.026 | 0.071013 | 0.171317 | <.001 |
| *β*2 - No Parental HardshipxAge^2 (ref) | -0.098 | 0.005 | -0.10816 | -0.08863 | <.001 | -0.089 | 0.007 | -0.10226 | -0.07644 | <.001 |
| *β*3 - No Parental HardshipxAge^3 (ref) | 0.004 | 0.0006 | 0.003012 | 0.005172 | <.001 | 0.003 | 0.0007 | 0.00108 | 0.003978 | 0.001 |
| *β*4 - No Parental HardshipxAge^4 (ref) | 0.002 | 0.000 | 0.001559 | 0.001968 | <.001 | 0.002 | 0.000 | 0.001251 | 0.001788 | <.001 |
| *β*5 - Parental Hardship Intercept | 0.816 | 0.179 | 0.465793 | 1.166097 | <.001 | 1.076 | 0.224 | 0.637344 | 1.514263 | <.001 |
| *β*6 - Parental HardshipxAge | 0.074 | 0.048 | -0.02126 | 0.16839 | 0.128 | -0.069 | 0.058 | -0.18391 | 0.044933 | 0.234 |
| *β*7 - Parental HardshipxAge^2 | -0.007 | 0.012 | -0.0314 | 0.016694 | 0.549 | -0.039 | 0.015 | -0.06946 | -0.00874 | 0.012 |
| *β*8 - Parental HardshipxAge^3 | -0.0010 | 0.0014 | -0.00372 | 0.001665 | 0.454 | 0.0035 | 0.0017 | 0.000118 | 0.006826 | 0.042 |
| *β*9 - Parental HardshipxAge^4 | 1E-04 | 0.000255 | -0.00036 | 0.000637 | 0.588 | 8E-04 | 0.000323 | 0.000153 | 0.001417 | 0.015 |
| Deviance | 208381.22 | | | | | 111617.6 | | | | |
| *Adjusted for child sex, parental age at birth, drinking in pregnancy, smoking in pregnancy, postnatal depression and physical abuse | | | | | | | |  |  |  |
| *Ref is the reference category which should be added to each category | | | |  |  |  |  |  |  |  |
| *Intercepts are at age 18 to coincide with the end of adolescence | |  |  |  |  |  |  |  |  |  |

| *Table S25A: Random effects of Parental Hardship on SMFQ Trajectories using Inverse Probability Weighting (IPW)* | | | | | | | | |
| --- | --- | --- | --- | --- | --- | --- | --- | --- |
|  | Maternal IPW Model (n=7412) | | | | Paternal IPW Model (n=3734) | | | |
| Parameter | Estimate | Std. Error | Low 95% CIs | High 95% CIs | Estimate | Std. Error | Low 95% CIs | High 95% CIs |
| var(Intercept) | 17.38325 | 0.548679 | 16.30786 | 18.45864 | 16.81522 | 0.758218 | 15.32914 | 18.3013 |
| cov(Intercept, Age) | 0.437369 | 0.100373 | 0.240642 | 0.634096 | 0.539128 | 0.13531 | 0.273925 | 0.804331 |
| var(Age) | 0.390562 | 0.035743 | 0.320507 | 0.460617 | 0.420227 | 0.047055 | 0.328001 | 0.512454 |
| cov(Intercept, Age^2) | -0.46617 | 0.03064 | -0.52622 | -0.40612 | -0.4641 | 0.04074 | -0.54395 | -0.38425 |
| cov(Age, Age^2) | 0.022318 | 0.007193 | 0.008219 | 0.036416 | 0.015653 | 0.009038 | -0.00206 | 0.033367 |
| var(Age^2) | 0.026514 | 0.002689 | 0.021243 | 0.031784 | 0.024719 | 0.003362 | 0.01813 | 0.031307 |
| cov(Intercept, Age^3) | 0.007009 | 0.002923 | 0.00128 | 0.012738 | 0.004145 | 0.003841 | -0.00338 | 0.011672 |
| cov(Age, Age^3) | -0.00714 | 0.000967 | -0.00904 | -0.00525 | -0.00793 | 0.001264 | -0.01041 | -0.00545 |
| cov(Age^2, Age^3) | -0.00073 | 0.000213 | -0.00115 | -0.00031 | -0.00057 | 0.000265 | -0.00109 | -5.3E-05 |
| var(Age^3) | 0.000186 | 2.79E-05 | 0.000132 | 0.000241 | 0.000203 | 3.57E-05 | 0.000133 | 0.000273 |
| cov(Intercept, Age^4) | 6.00E-03 | 5.86E-04 | 0.004849 | 0.007145 | 0.0059 | 7.63E-04 | 0.004405 | 0.007395 |
| cov(Age, Age^4) | -6.02E-04 | 1.53E-04 | -0.0009 | -0.0003 | -5.14E-04 | 1.87E-04 | -0.00088 | -0.00015 |
| cov(Age^2, Age^4) | -0.00036 | 5.25E-05 | -0.00046 | -0.00026 | -3.20E-04 | 6.47E-05 | -0.00045 | -0.00019 |
| cov(Age^3, Age^4) | 1.98E-05 | 4.52E-06 | 1.09E-05 | 2.86E-05 | 1.74E-05 | 5.47E-06 | 6.73E-06 | 2.82E-05 |
| var(Age^4) | 5.23E-06 | 1.10E-06 | 3.08E-06 | 7.38E-06 | 4.32E-06 | 1.34E-06 | 1.70E-06 | 6.94E-06 |

| *Table S26: Main Effects of Parental Personality x Parental Education x Time on SMFQ Trajectories using Inverse Probability Weighting (IPW)* | | | | | | | | | | |
| --- | --- | --- | --- | --- | --- | --- | --- | --- | --- | --- |
|  | Maternal IPW Model (n=6152) | | | | | Paternal IPW Model (n=2009) | | | | |
| Parameter | Estimate | Low 95% CIs | High 95% CIs | Std. Error | *p*-value | Estimate | Low 95% CIs | High 95% CIs | Std. Error | *p*-value |
| *β*0 - No PersonaliltyxHigher Education Intercept (ref) | 5.505333 | 0.304326 | 4.908865 | 6.1018 | <.001 | 5.619866 | 0.333267 | 4.966675 | 6.273057 | <.001 |
| *β*1 - No PersonaliltyxHigher EducationxAge (ref) | 0.049618 | 0.025458 | -0.00028 | 0.099516 | 0.051 | 0.090286 | 0.03802 | 0.015767 | 0.164804 | 0.018 |
| *β*2 - No PersonaliltyxHigher EducationxAge^2 (ref) | -0.08096 | 0.006567 | -0.09383 | -0.06809 | <.001 | -0.08767 | 0.009469 | -0.10623 | -0.06911 | <.001 |
| *β*3 - No PersonaliltyxHigher EducationxAge^3 (ref) | 0.004321 | 0.000746 | 0.002859 | 0.005782 | <.001 | 0.003639 | 0.001126 | 0.001432 | 0.005845 | 0.001 |
| *β*4 - No PersonaliltyxHigher EducationxAge^4 (ref) | 0.001454 | 0.000139 | 0.001182 | 0.001726 | <.001 | 0.001483 | 0.000199 | 0.001093 | 0.001874 | <.001 |
| *β*5 - PersonalityxLower Education Intercept | 1.619024 | 0.323582 | 0.984814 | 2.253234 | <.001 | 1.097202 | 0.676704 | -0.22911 | 2.423517 | 0.105 |
| *β*6 - PersonalityxLower EducationxAge | 0.232533 | 0.082307 | 0.071214 | 0.393851 | 0.005 | -0.26096 | 0.195706 | -0.64454 | 0.122612 | 0.182 |
| *β*7 - PersonalityxLower EducationxAge^2 | -0.05595 | 0.021675 | -0.09843 | -0.01347 | 0.01 | -0.0884 | 0.039521 | -0.16586 | -0.01094 | 0.025 |
| *β*8 - PersonalityxLower EducationxAge^3 | -0.00455 | 0.002392 | -0.00924 | 0.000139 | 0.057 | 0.005553 | 0.006275 | -0.00675 | 0.017851 | 0.376 |
| *β*9 - PersonalityxLower EducationxAge^4 | 0.000666 | 0.000462 | -0.00024 | 0.001571 | 0.149 | 0.001377 | 0.000952 | -0.00049 | 0.003243 | 0.148 |
| *β*10 - PersonalityxHigher Education Intercept | 0.340863 | 0.32013 | -0.28658 | 0.968306 | 0.287 | 0.098843 | 0.49552 | -0.87236 | 1.070045 | 0.842 |
| *β*11 - PersonalityxHigher EducationxAge | 0.055192 | 0.094802 | -0.13062 | 0.241001 | 0.56 | -0.08184 | 0.113586 | -0.30446 | 0.140787 | 0.471 |
| *β*12 - PersonalityxHigher EducationxAge^2 | -0.00162 | 0.023211 | -0.04711 | 0.043872 | 0.944 | 0.005095 | 0.041387 | -0.07602 | 0.086212 | 0.902 |
| *β*13 - PersonalityxHigher EducationxAge^3 | -4.6E-05 | 0.002713 | -0.00536 | 0.005271 | 0.986 | 0.002682 | 0.003502 | -0.00418 | 0.009545 | 0.444 |
| *β*14 - PersonalityxHigher EducationxAge^4 | 0.00015 | 0.000489 | -0.00081 | 0.001108 | 0.759 | 0.000166 | 0.0008 | -0.0014 | 0.001734 | 0.836 |
| *β*15 - No PersonaliltyxLower Education Intercept | 0.364733 | 0.150099 | 0.070545 | 0.658922 | 0.015 | -0.31591 | 0.251207 | -0.80827 | 0.176443 | 0.209 |
| *β*16 - No PersonaliltyxLower EducationxAge | 0.039634 | 0.038837 | -0.03648 | 0.115754 | 0.307 | -0.02136 | 0.063954 | -0.14671 | 0.103987 | 0.738 |
| *β*17 - No PersonaliltyxLower EducationxAge^2 | -0.03111 | 0.010258 | -0.05122 | -0.01101 | 0.002 | -0.00733 | 0.016488 | -0.03965 | 0.024987 | 0.657 |
| *β*18 - No PersonaliltyxLower EducationxAge^3 | -0.00007 | 0.001153 | -0.00233 | 0.00219 | 0.952 | 0.00065 | 0.001898 | -0.00307 | 0.004371 | 0.732 |
| *β*19 - No PersonaliltyxLower EducationxAge^4 | 0.000588 | 0.000216 | 0.000165 | 0.001011 | 0.006 | 0.000331 | 0.000348 | -0.00035 | 0.001012 | 0.342 |
| Deviance | 184745.79 | | | | | 66788.04 | | | | |
| *Adjusted for child sex, parental age at birth, drinking in pregnancy, smoking in pregnancy, postnatal depression and physical abuse | | | | | | | |  |  |  |
| *Ref is the reference category which should be added to each category | | |  |  |  |  |  |  |  |  |
| *Intercepts are at age 18 to coincide with the end of adolescence |  |  |  |  |  |  |  |  |  |  |

| *Table S26A: Random effects of Parental Personality x Parental Education x Time on SMFQ Trajectories using Inverse Probability Weighting (IPW)* | | | | | | | | |
| --- | --- | --- | --- | --- | --- | --- | --- | --- |
|  | Maternal IPW Model (n=6152) | | | | Paternal IPW Model (n=2009) | | | |
| Parameter | Estimate | Std. Error | Low 95% CIs | High 95% CIs | Estimate | Std. Error | Low 95% CIs | High 95% CIs |
| var(Intercept) | 17.24206 | 0.591921 | 16.08192 | 18.4022 | 16.67716 | 0.960779 | 14.79407 | 18.56025 |
| cov(Intercept, Age) | 0.469942 | 0.105017 | 0.264113 | 0.675771 | 0.485651 | 0.170758 | 0.15097 | 0.820331 |
| var(Age) | 0.371855 | 0.037146 | 0.29905 | 0.44466 | 0.392355 | 0.056656 | 0.281311 | 0.503399 |
| cov(Intercept, Age^2) | -0.46377 | 0.032889 | -0.52823 | -0.39931 | -0.44032 | 0.051757 | -0.54176 | -0.33888 |
| cov(Age, Age^2) | 0.02166 | 0.007563 | 0.006837 | 0.036484 | 0.011024 | 0.010878 | -0.0103 | 0.032345 |
| var(Age^2) | 0.026128 | 0.002808 | 0.020625 | 0.031631 | 0.021727 | 0.00394 | 0.014005 | 0.029449 |
| cov(Intercept, Age^3) | 0.006582 | 0.003102 | 0.000504 | 0.012661 | 0.005665 | 0.004952 | -0.00404 | 0.015371 |
| cov(Age, Age^3) | -0.00667 | 0.00101 | -0.00865 | -0.00469 | -0.00767 | 0.00156 | -0.01073 | -0.00462 |
| cov(Age^2, Age^3) | -0.00073 | 0.000225 | -0.00117 | -0.00029 | -0.00049 | 0.000323 | -0.00113 | 0.000137 |
| var(Age^3) | 0.000176 | 2.94E-05 | 0.000119 | 0.000234 | 0.000218 | 0.000046 | 0.000127 | 0.000308 |
| cov(Intercept, Age^4) | 0.005953 | 0.000627 | 0.004724 | 0.007182 | 0.005529 | 0.000984 | 0.0036 | 0.007457 |
| cov(Age, Age^4) | -0.00059 | 0.000161 | -0.00091 | -0.00028 | -0.00043 | 0.000226 | -0.00087 | 1.47E-05 |
| cov(Age^2, Age^4) | -0.00036 | 0.000055 | -0.00046 | -0.00025 | -0.00027 | 7.54E-05 | -0.00042 | -0.00012 |
| cov(Age^3, Age^4) | 0.00002 | 4.79E-06 | 1.06E-05 | 2.94E-05 | 1.75E-05 | 6.68E-06 | 4.39E-06 | 3.06E-05 |
| var(Age^4) | 5.20E-06 | 1.16E-06 | 2.94E-06 | 7.47E-06 | 3.63E-06 | 1.55E-06 | 5.91E-07 | 6.67E-06 |

| *Table S27: Random effects of Parental Personality x Parental Hardship (PHA) x Time on SMFQ Trajectories using Inverse Probability Weighting (IPW)* | | | | | | | | | | |
| --- | --- | --- | --- | --- | --- | --- | --- | --- | --- | --- |
|  | Maternal IPW Model (n=6021) | | | | | Paternal IPW Model (n=1960) | | | | |
| Parameter | Estimate | Low 95% CIs | High 95% CIs | Std. Error | *p*-value | Estimate | Low 95% CIs | High 95% CIs | Std. Error | *p*-value |
| *β*0 - No PersonaliltyxNo PHA Intercept (ref) | 5.472261 | 0.284537 | 4.914578 | 6.029944 | <.001 | 5.31174 | 0.334282 | 4.656559 | 5.966921 | <.001 |
| *β*1 - No PersonaliltyxNo PHAxAge (ref) | 0.062713 | 0.02069 | 0.02216 | 0.103265 | 0.002 | 0.13608 | 0.033665 | 0.070098 | 0.202063 | <.001 |
| *β*2 - No PersonaliltyxNo PHAxAge^2 (ref) | -0.09628 | 0.005531 | -0.10712 | -0.08544 | <.001 | -0.08361 | 0.008491 | -0.10025 | -0.06696 | <.001 |
| *β*3 - No PersonaliltyxNo PHAxAge^3 (ref) | 0.004222 | 0.000616 | 0.003014 | 0.00543 | <.001 | 0.001675 | 0.000982 | -0.00025 | 0.0036 | 0.088 |
| *β*4 - No PersonaliltyxNo PHAxAge^4 (ref) | 0.001718 | 0.000116 | 0.00149 | 0.001946 | <.001 | 0.001363 | 0.000176 | 0.001017 | 0.001709 | <.001 |
| *β*5 - PersonalityxNo PHA Intercept | 0.736103 | 0.270457 | 0.206017 | 1.266188 | 0.006 | 0.114641 | 0.456265 | -0.77962 | 1.008903 | 0.802 |
| *β*6 - PersonalityxNo PHAxAge | 0.090168 | 0.075383 | -0.05758 | 0.237916 | 0.232 | -0.25343 | 0.10102 | -0.45143 | -0.05544 | 0.012 |
| *β*7 - PersonalityxNo PHAxAge^2 | -0.02044 | 0.019387 | -0.05844 | 0.017554 | 0.292 | -0.04795 | 0.037993 | -0.12242 | 0.026513 | 0.207 |
| *β*8 - PersonalityxNo PHAxAge^3 | -0.00124 | 0.002235 | -0.00562 | 0.003144 | 0.58 | 0.010264 | 0.003571 | 0.003265 | 0.017264 | 0.004 |
| *β*9 - PersonalityxNo PHAxAge^4 | 0.000347 | 0.00042 | -0.00048 | 0.00117 | 0.409 | 0.00163 | 0.000767 | 0.000127 | 0.003134 | 0.034 |
| *β*10 - PersonalityxYes PHA Intercept | 1.613466 | 0.433407 | 0.764004 | 2.462929 | <.001 | 2.063282 | 0.791499 | 0.511972 | 3.614592 | 0.009 |
| *β*11 - PersonalityxYes PHAxAge | 0.26684 | 0.109295 | 0.052627 | 0.481053 | 0.015 | 0.028336 | 0.236548 | -0.43529 | 0.491961 | 0.905 |
| β12 - PersonalityxYes PHAxAge^2 | -0.01395 | 0.027828 | -0.06849 | 0.040589 | 0.616 | -0.01023 | 0.04461 | -0.09767 | 0.077201 | 0.819 |
| β13 - PersonalityxYes PHAxAge^3 | -0.00579 | 0.00296 | -0.0116 | 8.35E-06 | 0.05 | -0.00301 | 0.006651 | -0.01605 | 0.010022 | 0.65 |
| β14 - PersonalityxYes PHAxAge^4 | -0.0001 | 0.000553 | -0.00119 | 0.000983 | 0.854 | -0.00069 | 0.000954 | -0.00256 | 0.001183 | 0.471 |
| β15 - No PersonaliltyxYes PHA Intercept | 0.739623 | 0.218721 | 0.310937 | 1.168308 | 0.001 | 1.08515 | 0.336415 | 0.42579 | 1.74451 | 0.001 |
| β16 - No PersonaliltyxYes PHAxAge | 0.038735 | 0.058592 | -0.0761 | 0.153573 | 0.509 | -0.26309 | 0.084124 | -0.42797 | -0.09821 | 0.002 |
| β17 - No PersonaliltyxYes PHAxAge^2 | -0.00794 | 0.015108 | -0.03755 | 0.021673 | 0.599 | -0.05574 | 0.021963 | -0.09879 | -0.0127 | 0.011 |
| β18 - No PersonaliltyxYes PHAxAge^3 | 0.000329 | 0.001712 | -0.00303 | 0.003686 | 0.847 | 0.009954 | 0.002542 | 0.004971 | 0.014936 | 0 |
| β19 - No PersonaliltyxYes PHAxAge^4 | 0.000276 | 0.000316 | -0.00034 | 0.000895 | 0.381 | 0.001379 | 0.000466 | 0.000465 | 0.002292 | 0.003 |
| Deviance | 180998.65 | | | | | 65099.58 | | | | |
| *Adjusted for child sex, paternal age at birth, drinking in pregnancy, smoking in pregnancy, postnatal depression and physical abuse | | | | | | | |  |  |  |
| *Ref is the reference category which should be added to each category | | | |  |  |  |  |  |  |  |
| *Intercepts are at age 18 to coincide with the end of adolescence | |  |  |  |  |  |  |  |  |  |

| *Table S27A: Random effects of Parental Personality x Parental Hardship x Time on SMFQ Trajectories using Inverse Probability Weighting (IPW)* | | | | | | | | |
| --- | --- | --- | --- | --- | --- | --- | --- | --- |
|  | Maternal IPW Model (n=6021) | | | | Paternal IPW Model (n=1960) | | | |
| Parameter | Estimate | Std. Error | Low 95% CIs | High 95% CIs | Estimate | Std. Error | Low 95% CIs | High 95% CIs |
| var(Intercept) | 17.24936 | 0.604908 | 16.06376 | 18.43496 | 16.86982 | 0.987533 | 14.9343 | 18.80535 |
| cov(Intercept, Age) | 0.476484 | 0.106669 | 0.267416 | 0.685551 | 0.56958 | 0.174888 | 0.226807 | 0.912354 |
| var(Age) | 0.368535 | 0.037318 | 0.295393 | 0.441677 | 0.392059 | 0.056737 | 0.280857 | 0.503261 |
| cov(Intercept, Age^2) | -0.46734 | 0.033355 | -0.53271 | -0.40196 | -0.46642 | 0.051785 | -0.56791 | -0.36492 |
| cov(Age, Age^2) | 0.020388 | 0.007593 | 0.005506 | 0.03527 | 0.006562 | 0.010851 | -0.01471 | 0.02783 |
| var(Age^2) | 0.02617 | 0.002848 | 0.020589 | 0.031751 | 0.022974 | 0.003977 | 0.015179 | 0.030768 |
| cov(Intercept, Age^3) | 0.005818 | 0.003141 | -0.00034 | 0.011974 | 0.002658 | 0.004968 | -0.00708 | 0.012395 |
| cov(Age, Age^3) | -0.00661 | 0.00101 | -0.00859 | -0.00463 | -0.00756 | 0.001542 | -0.01059 | -0.00454 |
| cov(Age^2, Age^3) | -0.00067 | 0.000225 | -0.00112 | -0.00023 | -0.00039 | 0.000318 | -0.00101 | 0.000237 |
| var(Age^3) | 0.000174 | 2.93E-05 | 0.000116 | 0.000231 | 0.000211 | 4.47E-05 | 0.000124 | 0.000299 |
| cov(Intercept, Age^4) | 0.005942 | 0.000635 | 0.004699 | 0.007186 | 0.005831 | 0.000977 | 0.003917 | 0.007745 |
| cov(Age, Age^4) | -0.00057 | 0.000161 | -0.00088 | -0.00025 | -0.00034 | 0.000223 | -0.00077 | 9.89E-05 |
| cov(Age^2, Age^4) | -0.00035 | 5.56E-05 | -0.00046 | -0.00024 | -0.00028 | 7.57E-05 | -0.00043 | -0.00013 |
| cov(Age^3, Age^4) | 1.89E-05 | 4.79E-06 | 9.55E-06 | 2.83E-05 | 1.53E-05 | 6.54E-06 | 2.49E-06 | 2.81E-05 |
| var(Age^4) | 5.07E-06 | 1.16E-06 | 2.79E-06 | 7.35E-06 | 3.62E-06 | 1.55E-06 | 5.81E-07 | 6.66E-06 |
